# Comparing machine learning classifier models in discriminating cognitively unimpaired older adults from three clinical cohorts in the Alzheimer’s disease spectrum: demonstration analyses in the COMPASS-ND study

**DOI:** 10.3389/fnagi.2025.1542514

**Published:** 2025-03-04

**Authors:** Harrison Fah, Linzy Bohn, Russell Greiner, Roger A. Dixon

**Affiliations:** ^1^Neuroscience and Mental Health Institute, University of Alberta, Edmonton, AB, Canada; ^2^Department of Computing Science, University of Alberta, Edmonton, AB, Canada; ^3^Department of Psychology, University of Alberta, Edmonton, AB, Canada; ^4^Department of Psychiatry, University of Alberta, Edmonton, AB, Canada; ^5^Alberta Machine Intelligence Institute, Edmonton, AB, Canada

**Keywords:** Alzheimer’s disease, Artificial Intelligence, Canadian consortium on neurodegeneration in aging, machine learning, mild cognitive impairment, eXplainable Artificial Intelligence

## Abstract

**Background:**

Research in aging, impairment, and Alzheimer’s disease (AD) often requires powerful computational models for discriminating between clinical cohorts and identifying early biomarkers and key risk or protective factors. Machine Learning (ML) approaches represent a diverse set of data-driven tools for performing such tasks in big or complex datasets. We present systematic demonstration analyses to compare seven frequently used ML classifier models and two eXplainable Artificial Intelligence (XAI) techniques on multiple performance metrics for a common neurodegenerative disease dataset. The aim is to identify and characterize the best performing ML and XAI algorithms for the present data.

**Method:**

We accessed a Canadian Consortium on Neurodegeneration in Aging dataset featuring four well-characterized cohorts: Cognitively Unimpaired (CU), Subjective Cognitive Impairment (SCI), Mild Cognitive Impairment (MCI), and AD (*N* = 255). All participants contributed 102 multi-modal biomarkers and risk factors. Seven ML algorithms were compared along six performance metrics in discriminating between cohorts. Two XAI algorithms were compared using five performance and five similarity metrics.

**Results:**

Although all ML models performed relatively well in the extreme-cohort comparison (CU/AD), the Super Learner (SL), Random Forest (RF) and Gradient-Boosted trees (GB) algorithms excelled in the challenging near-cohort comparisons (CU/SCI). For the XAI interpretation comparison, SHapley Additive exPlanations (SHAP) generally outperformed Local Interpretable Model agnostic Explanation (LIME) in key performance properties.

**Conclusion:**

The ML results indicate that two tree-based methods (RF and GB) are reliable and effective as initial models for classification tasks involving discrete clinical aging and neurodegeneration data. In the XAI phase, SHAP performed better than LIME due to lower computational time (when applied to RF and GB) and incorporation of feature interactions, leading to more reliable results.

## 1 Introduction

Alzheimer’s Disease (AD), the most common cause of dementia, is a complex neurodegenerative disease associated with progressive accumulation of characteristic neuropathology (neurofibrillary tangles and amyloid plaques), atrophy of cortex (including hippocampus), and reduced memory and cognitive performance, which in turn degrades the ability to perform daily tasks and activities (Alzheimer’s Association, 2023). Reviews and meta-analyses of observational studies have shown that pathways toward AD are associated with biomarkers and risk factors representing multiple domains of aging systems (Anstey et al., 2019; Dixon and Lachman, 2019; Livingston et al., 2020). A diagnosis of AD is frequently preceded by classifiable conditions such as Mild Cognitive Impairment (MCI), which is signaled by objective cognitive impairment without dementia (Petersen, 2004), and Subjective Cognitive Impairment (SCI), which is indicated by self-reports of subjective cognitive complaints and concerns in the absence of objective signs of cognitive impairment (Jessen et al., 2020). Compared to those that are Cognitively Unimpaired (CU), persons classified as having SCI or MCI are at an elevated risk of exacerbated cognitive decline and conversion to AD (Koppara et al., 2015; Slot et al., 2018). The typical long, complex, and multi-faceted pre-diagnosis onset period associated with AD and Related Disorders (ADRD) presents unique challenges for research aimed at detecting leading characteristics of early dementia risk. However, the recent concomitant emergence of large-scale databases and development of advanced data analytic techniques have demonstrated considerable promise for addressing these challenges (Badhwar et al., 2020; Hampel et al., 2019; Iturria-Medina et al., 2016). Furthermore, recent reviews have expressed the importance of assembling multi-dimensional databases for investigating which features (or combinations thereof) can be used to (1) detect early and intensifying AD risk, (2) discriminate among AD and related neurodegenerative disorders, and (3) identify crucial stratification factors (e.g., sex, genetic risk) (Quiñones et al., 2020; Yarnall et al., 2017). Accordingly, Artificial Intelligence (AI) has provided a framework for developing, testing, and deploying data-driven analytic techniques that systematically search and detect patterns and useful associations within such large, high-dimensional, and even dynamic (longitudinal) datasets (Zhou et al., 2017). In the present article, we provide a focused review of general Machine Learning (ML) algorithms and specific ML models of relevance to the field. Using a high-dimensional aging and AD-related dataset, we (1) assemble seven prominent supervised ML classifier approaches, (2) identify key metrics for evaluating basic model prediction performance, (3) select two prominent follow-up AI-based explanation protocols, and (4) compute independent results, evaluate relative performance, and determine the leading predictors.

ML, a subfield of AI, has been increasingly used in studies detecting early AD risk factors and patterns, characterizing heterogeneous preclinical trajectories, discriminating among AD and related neurodegenerative conditions, precision diagnosis of AD and neurodegenerative subtypes, and even identifying personalized therapeutic options (Fathi et al., 2022; Pellegrini et al., 2018). ML methods include both unsupervised and supervised learning. The latter is the most common method of ML used in neurodegeneration research (Myszczynska et al., 2020). It involves a computational model learning the relationship between a set of features (e.g., age, sex, education, genetics) and a label (e.g., MCI). The model learns by studying a set of examples in its training dataset and can then be used to predict the labels (e.g., latent classes, subgroups) of unseen samples. ML offers advantages over traditional statistical analysis methods due to its ability to process multiple variables of various types (e.g., imaging, cognition, clinical) and formats (e.g., discrete, continuous, categorical), as well as produce effective models using both small and large datasets. Notably, given the multifactorial and progressive nature of neurodegenerative diseases, ML applications have proven capable of dealing with multiple modalities of information such as neuroimaging, biological markers, genomic, demographic, metabolic, omics-related, morbidities, lifestyle, cognition, and a variety of risk-related exposures (Tanveer et al., 2020).

However, the rapid emergence and growing availability of a variety of powerful ML techniques have created both opportunities and challenges for researchers in the field. Active questions include for a given clinical aging and neurodegeneration dataset (1) whether one or a combination of ML techniques is preferred, (2) how to evaluate and compare the relative performance of leading ML techniques, and (3) how to incorporate and compare eXplainable Artificial Intelligence (XAI) techniques for follow-up interpretation. These questions are of immediate relevance, as it is becoming increasingly common for researchers to include more than one ML technique in their analyses (e.g., Badhwar et al., 2020; Bloch and Friedrich, 2021; Bohn et al., 2023; Drouin et al., 2022; McFall et al., 2023). At present, the criteria for selecting which model to employ and how to determine relative performance differences among the models are unclear.

A common goal of ML applications in neurodegeneration is to determine feature importance, which refers to the degree to which a feature influences a model’s prediction. However, some ML methods are referred to as “black-box” approaches, in that they identify important features but do not provide deeper interpretative guidelines. Following identification of important features, a deeper analysis can be applied to any ML predictive model to reveal key clinically and theoretically important aspects of the results. These XAI methods can be integrated with a supervised ML model in a two-step process. First, a model is trained on a dataset of well-characterized and labeled individuals with a large set of features possibly related to the condition being predicted. Second, a post-hoc XAI algorithm is applied to the model to calculate feature importance values that can be used to rank features by their influence either on local (i.e., individual) or global (e.g., clinical subgroup) predictions. Recent studies have used different combinations of supervised ML and XAI algorithms, typically evaluating results with the help of domain experts and finding correspondences within existing neurodegenerative disease literature (Bellantuono et al., 2022; Bohn et al., 2023; El-Sappagh et al., 2021; McFall et al., 2023; Sudar et al., 2022). However, an important challenge facing researchers interested in applying these data-driven ML approaches to neurodegeneration databases is the formidable range of both supervised ML algorithms and XAI techniques, each of which has unique characteristics that may perform differentially across combinations of predictors, conditions, diseases, and datasets (Ellis et al., 2022). The overall aim of the present study is to systematically evaluate and compare the performance (e.g., accuracy, precision) of seven prominent supervised ML classification algorithms and relevant properties (e.g., runtime, distributions of importance values) of two complementary XAI interpretation techniques as applied to a common dataset with cohorts representing formally classified phases along the AD spectrum. We compare the performance of these supervised ML models using six commonly used ML metrics. We compare the XAI algorithms using five independently derived metrics describing performance and five metrics describing similarity between results.

For the demonstration analyses, we use the cohort database from the Canadian Consortium on Neurodegeneration in Aging (CCNA) study, which is referred to as the Comprehensive Assessment of Neurodegeneration and Dementia (COMPASS-ND) (Chertkow et al., 2019). The COMPASS-ND database includes a wide range of AD-related risk features potentially relevant to the current aim. At present, the dataset is “cross-sectional,” including only one occasion of measurement for all participants. We adapted the feature protocol of a previous study (Bohn et al., 2023) and selected a set of 102 indicators of multiple morbidities and deficits (Kernick et al., 2017) from 17 domains (e.g., biomarkers, quality of life, diseases, physical activity, sleep, frailty). Morbidities increase in number and severity with aging and are linked with adverse outcomes such as accelerated cognitive decline, impairment, dementia, institutionalization, and death (Bohn et al., 2023; Grande et al., 2019; Kernick et al., 2017; Kojima et al., 2016; Koyanagi et al., 2018; Thibeau et al., 2019; Ward et al., 2021; Wei et al., 2020). The associated risks of neurodegeneration from multiple morbidities indicates a promising approach for identifying key morbidities that (1) discriminate CU older adults from those with SCI, MCI, or AD, and (2) point to potential early indicators of elevated AD risk.

We examine three specific goals. For Research Goal (RG) 1, we calculated and compared the performance qualities of seven common and promising ML algorithms used in neurodegeneration and related research for discriminating between cohorts over four classification tasks (three binary and one simultaneous multi-class). We examined the ML algorithms by considering six model performance indicators separated into two clusters as described above (primary and secondary). The metrics are further described below.

For RG 2, we examined the relative performance and similarity of two XAI model interpretation algorithms by comparing them according to five independently derived performance metrics (separated into primary and secondary clusters as described above) and five directly comparative similarity metrics. The performance and similarity metrics are further described below.

For RG 3, we informally compared the ML-XAI models in terms of the similarity of identified sets of leading morbidity-related features within the pairwise cohort comparisons.

We expect these comprehensive comparative analyses to provide methodological and practical insights into which ML and XAI algorithms and indicators perform best and can be recommended for brain aging and dementia datasets with variables and objectives similar to those in this study. We also expect this study to supplement ongoing research on multi-modal biomarkers or morbidities that are potentially significant in the prediction of disorders related to and including AD (Bohn et al., 2023; McFall et al., 2023; Sapkota and Dixon, 2018).

## 2 Methods

### 2.1 Database and participants

The CCNA-based COMPASS-ND study participants were recruited in 31 data collection sites across Canada with coordinated ethics approval from the Research Ethics Board of each participating site and written informed consent from all participants. A detailed methodological summary of the study has been published in Chertkow et al. (2019). Exclusionary criteria in the COMPASS-ND study protocol were: (1) presence of significant known chronic brain disease, multiple sclerosis, a serious developmental handicap, malignant tumors, Huntington’s disease, and other rarer brain illnesses; (2) ongoing drug or alcohol abuse; (3) total score < 13 on the Montreal Cognitive Assessment (Nasreddine et al., 2005); (4) symptomatic stroke within the previous year; or (5) unwilling or unable to undergo magnetic resonance imaging scans. Eligible participants were (1) sufficiently proficient in English or French and (2) had a study partner that they interacted with weekly. Participants were formally classified or diagnosed, depending on the condition, by consensus among expert clinician researchers involved in the CCNA (Chertkow et al., 2019). Therefore, the classification of participants in the SCI and MCI categories and the diagnosis of participants with AD were conducted independently and prior to the present study. Accordingly, no classifications or diagnoses were produced in the present study or performed with the assistance of ML techniques. For the present study, we examine the cross-sectional dataset and excluded individuals outside the AD spectrum, specifically those with a diagnosis of subcortical ischemic vascular MCI, dementia of mixed etiology, frontotemporal dementia, Parkinson’s disease, and Lewy body dementia. The final study sample (*N* = 255; M age = 71.18; 58% female; 92% non-Hispanic White) was comprised of four cohorts who varied in clinical severity along an AD spectrum: CU (*n* = 60), SCI (*n* = 36), MCI (*n* = 116), AD (*n* = 43) (Bohn et al., 2023). As a result of (1) the differential availability of participants from each cohort in COMPASS-ND and (2) our decision to retain as many participants as possible, there is an imbalance in the number of individuals across cohorts. Characteristics of the participants are summarized in Table 1.

**Table 1:** Demographic and clinical characteristics for each cohort.

Characteristic	CU (*n* = 60)	SCI (*n* = 36)	MCI (*n* = 116)	AD (*n* = 43)	Significance
*n* (%) female	49 (82%)^a^	30 (83%)^a^	57 (49%)^b^	13 (30%)^c^	***
Age in years	69.23 (5.52)^a^	69.62 (6.81)^a^	71.16 (6.48)^a^	75.26 (7.70)^b^	***
Education in years	15.84 (3.15)	17.49 (3.11)	15.75 (3.89)	15.34 (4.37)	ns
*n* (%) married	37 (62%)^a^	17 (47%)^a^	75 (65%)^a^	35 (81%)^b^	*
*n* (%) Non-Hispanic White	58 (97%)^a^	34 (94%)^a,b^	100 (86%)^b^	42 (98%)^a^	*
MoCA	27.90 (1.50)^a^	27.81 (1.33)^a^	24.28 (3.08)^b^	18.63 (3.56)^c^	***

Results are presented as mean (standard deviation) unless noted as otherwise. *p*- values are based on one-way analysis of variance or chi-square tests, as appropriate. We adjusted for multiple comparisons using post-hoc Tukey tests. CU, cognitively unimpaired; SCI, subjective cognitive impairment; MCI, mild cognitive impairment; AD, Alzheimer’s disease; sig, significance; ns, not significant; MoCA, Montreal Cognitive Assessment. ^a–c^Denotes values that differ significantly. *p- value < 0.05; ** *p*- value < 0.01; ***p- value < 0.001.

#### 2.1.1 Pool of predictive features: morbidity and deficit indicators

We used 102 indicators of morbidity and other aging deficits that were assembled and evaluated in a previous COMPASS-ND study (Bohn et al., 2023), all of which have been linked with adverse outcomes such as frailty, functional deficits, accelerated cognitive decline or impairment, neurodegenerative disease, and institutionalization (Ward et al., 2021). These indicators were selected according to expert recommendations and determined to represent the following 17 morbidity domains: instrumental activities of daily living (ADL), basic ADL, physical activity, mobility, quality of life (QoL), anthropometric measures, sensory function, sleep, functional performance, exhaustion, self-reported health, cardiorespiratory health, clinical symptoms or diseases, emotional well-being, oral health and nutritional factors, fluid biomarkers, and sex. These indicators were collected through self-report, physical examination, and formal tests with standardized scales. Indicators had values that ranged between 0 (no deficit recorded) and 1 (deficit is maximally expressed) (Searle et al., 2008). Consistent with previous research (Bohn et al., 2023), we removed indicators (1) where less than 10% of participants in each cohort were recorded as having the deficit and (2) with a rate of missingness >50% (Hassler et al., 2019; Madley-Dowd et al., 2019).

The four cross-sectional data subsets and final number of available features were as follows: SCI vs. CU (64), MCI vs. CU (65), AD vs. CU (75) and all four cohorts (56). The analyses included three binary discrimination tasks and one simultaneous four-way discrimination task. There were 83 unique features across all datasets. These features (disaggregated by domain) and their corresponding response scales are presented in Supplementary Table 1. Across the entire study sample, the rate of missingness for the final set of morbidity features ranged between 0 and 3%, with the average rate across predictors at 0.7%.

#### 2.1.2 Handling missing data

As noted above, missing values were rare across persons and features (M = 0.7%) in the COMPASS-ND dataset. ML base learner models use an imputer to fit on the training dataset and estimate missing values. We use the scitkit-learn IterativeImputer with a BayesianRidge estimator (Pedregosa et al., 2011). The Bayesian ridge regression model is used to estimate the missing feature as a function of the other features. This approach uses all the data points to estimate the missing value.

### 2.2 Seven machine learning algorithms

AI-informed, data-driven procedures such as supervised ML are preferred applications for problems such as predicting cognitive impairment as they learn correlations among multiple features and corresponding labels simultaneously. We selected seven supervised ML algorithms of notable relevance to aging and neurodegeneration and implemented them independently and comparatively in this study. We distinguished between two subclasses of algorithms, base and ensemble. These subclasses share the overall goal of building a classifier model—an algorithm that predicts the class of an input instance (expressed as a feature vector)—but differ according to algorithm implementation (i.e., steps by which the classifier is trained or generates a prediction). Base learner methods either implement a single algorithm or multiple homogenous algorithms (i.e., using multiple decision trees) in producing a classifier. The ML base learner subclass includes the following techniques: Logistic Regression (LR), Support Vector Machines (SVM), Random Forest (RF), Gradient-Boosted trees (GB), and Artificial Neural Networks (ANN) (Belić et al., 2019; Chang et al., 2021; Myszczynska et al., 2020; Tăuţan et al., 2021). Ensemble methods, on the other hand, integrate multiple base learners in a unified approach either by comparing their internal results and selecting the best model to use or by aggregating the predictions of multiple models to make a final prediction. The ensemble subclass includes Voting Ensembles (VE) (Dietterich, 2000) and Super Learners (SE) (van der Laan et al., 2007). We highlight the advantages and disadvantages of each ML algorithm in Table 2.

**Table 2:** Key consensus characteristics of the seven supervised ML algorithms.

ML algorithm	Advantages	Disadvantages
Logistic Regression (LR)	Simple Fast training Typically has few hyperparameters	Restricted to linear functions
Support Vector Machines (SVM)	Good generalization Can solve nonlinear problems using kernel functions	Performs poorly on noisy data (e.g., considerable overlap between classes)
Random Forest (RF)	Learns a nonlinear function Performs well on categorical data Less prone to overfitting	Less useful for linear problems Numerous hyperparameters
Gradient-Boosted trees (GB)	Learns a nonlinear function Performs well on categorical data	Less useful for linear problems Numerous hyperparameters Can suffer from overfitting
Artificial Neural Network (ANN)	Can learn both linear and nonlinear functions Robust to noisy data	Requires a lot of hyperparameter tuning Difficult to understand why a prediction was made
Voting Ensemble (VE)	Can include a variety of ML algorithms (e.g., LR, SVM, GB, ANN) Generalizes well	Slow training
Super Learner (SL)	Can compare a variety of ML algorithms such as the ones above Can select model and hyperparameters without human oversight	Slow training Only as good as the best performing base learner

Information adapted from Bentéjac et al. (2020), Chang et al. (2021).

#### 2.2.1 Logistic regression

LR is one of the most popular ML algorithms for classification across many fields due to its simplicity and interpretability (Boateng and Abaye, 2019; Dreiseitl and Ohno-Machado, 2002). LR is a linear classifier method as it generates the predicted probability of a class by applying either a sigmoid or softmax function to the weighed sum of the features in the input vector. These weights are learned during training by minimizing a loss function (e.g., log loss) over the training data via an optimization algorithm (e.g., gradient descent). We use scikit-learn’s LogisticRegression class for the LR model (Pedregosa et al., 2011).

#### 2.2.2 Support vector machine

Due to its simplicity, fast computation times, and good generalization performance, SVM is a widely used ML algorithm for classification and regression problems. It is also one of the most common approaches applied to aging and AD databases (Arya et al., 2023; Tanveer et al., 2020). SVMs are trained to find a function or decision boundary that separates points of two classes in a way that maximizes the width of the gap between them. To classify non-linear data, a kernel function can be used to transform the inputs to higher dimensions. In the multi-class scenario, a one-vs-one approach is used for training the model in which distinct classifiers are trained for each pair of classes and used together for the prediction. We used scikit-learn’s SVC class to implement the SVM (Pedregosa et al., 2011).

#### 2.2.3 Random forest

The RF algorithm derives its name from its use of many decision trees trained independently via random feature and sample selection (Gray et al., 2013). Decision trees work by generating predictions through a hierarchical rule-based approach. RF combines these many trees by averaging their predictions to improve overall accuracy and reduce overfitting. It has shown impressive results in predicting AD when trained on various datasets including neuroimaging and multi-modal data (Dimitriadis et al., 2018; Gray et al., 2013). RF also extends naturally to multi-class problems. While RF is an ensemble method, we refer to it as a base learner due to its use in the VE and SL algorithms. We used scikit-learn’s RandomForestClassifier class for the implementation of RF (Pedregosa et al., 2011).

#### 2.2.4 Gradient-boosted trees

Gradient boosting refers to an algorithm that utilizes weak base learners through boosting (i.e., iteratively improving the learners and adding them to the final classifier). In the case of GB, decision trees are used as the weak learner. Due to its iterative process, GB is vulnerable to overfitting if the hyperparameters are not properly set (Bentéjac et al., 2020). Like RF, we refer to GB as a base learner instead of an ensemble method for reasons noted above. We used scikit-learn’s GradientBoostingClassifier for the implementation of GB (Pedregosa et al., 2011).

#### 2.2.5 Artificial neural network

ANNs are designed similarly to biological neural networks and have recently seen substantial success in many different fields and applications (Abiodun et al., 2018). Their ability to learn non-linear associations makes them an attractive fit for complex data for which labels are likely determined by interactions of features. ANNs have been used with a wide range of modalities for predicting the presence of AD (Tanveer et al., 2020). ANNs are trained through the process of backpropagation where the loss from a prediction is sent back through the network to update the neurons. We implemented the ANN as a Multilayer Perceptron (MLP) using scikit-learn’s MLPClassifier class (Pedregosa et al., 2011).

#### 2.2.6 Voting ensemble

VE is a combination of multiple ML models in which the final prediction is determined by using the predictions from each of the models. The base learners can either be different models or the same model with different hyperparameters. There are typically two main types of voting: (1) soft, for which the probabilities from each model are added up and the class with the largest sum is selected; or (2) hard, for which each model casts a vote toward a class and the one with the most votes is selected. We used the scikit-learn VotingClassifier class, which is composed of LR, SVM, RF, GB, and ANN with soft voting for the VE (Pedregosa et al., 2011). As mentioned earlier, VE uses the hyperparameters selected for each model when computing the SHAP values on the entire dataset and thus the performance values may be inflated.

#### 2.2.7 Super learner

SL is similar to VE in that it combines multiple different ML models internally (van der Laan et al., 2007). However, SL combines them in the training process and only a single model is selected for use in making predictions after training. In our case, the model selected is the one with the highest internal cross-validation Area Under receiver-operating characteristic Curve (AUC). We used the five base learners: LR, SVM, RF, GB, and ANN. Each model was implemented using the scikit-learn class and the list of possible hyperparameters mentioned in the associated section above (Pedregosa et al., 2011).

### 2.3 Hypertuning and hyperparameters

**Figure 1:**
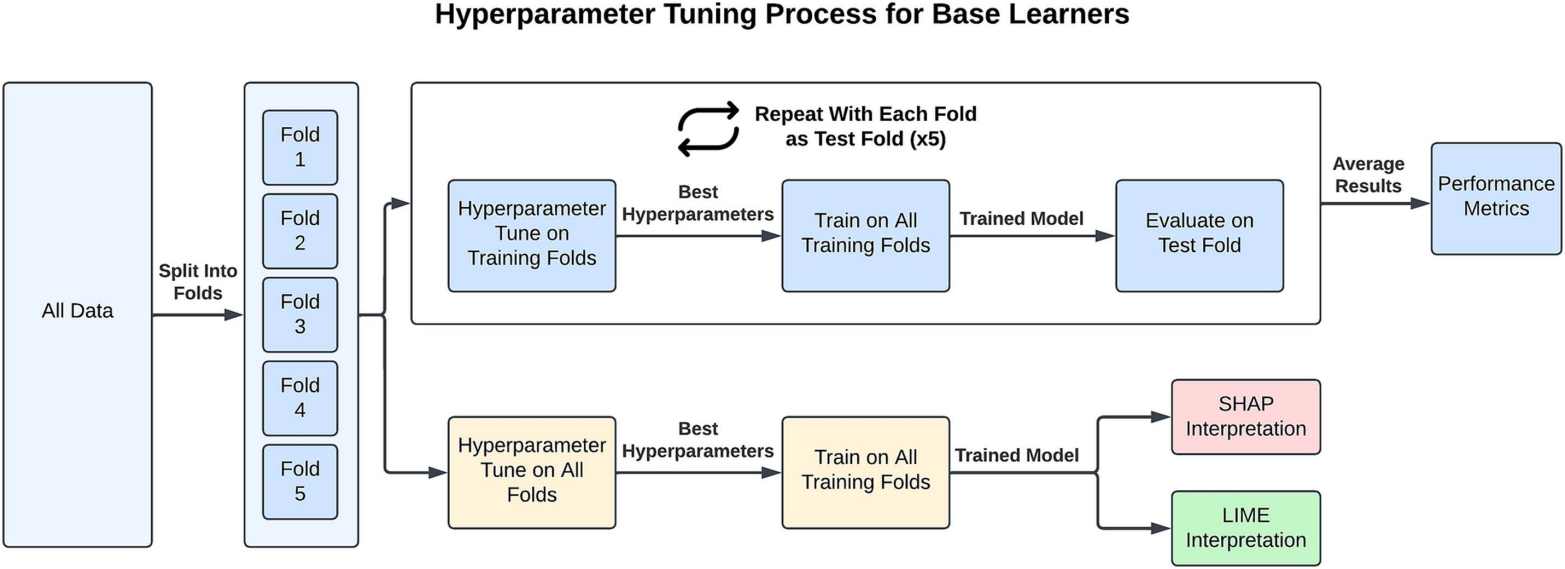
For each task, the hypertuning process for a base learner involves splitting the dataset into five folds. Four folds are used for training, where nested five-fold validation identifies the best hyperparameters. These hyperparameters are then applied to train a model on all training folds, and the model’s performance is evaluated on the test fold using six performance metrics. This process is repeated so that each fold is used as the test fold once, and the performance metrics are averaged across all five train/test fold combinations. For XAI interpretation, all folds are used to identify the best hyperparameters. A final model is then trained on all folds with these hyperparameters, and SHAP/LIME is applied for interpretability.

The choice of hyperparameters from each of the base learners can be found in Supplementary Table 2 and the top performing hyperparameters for each task are presented in Supplementary Tables 3–6. To select the optimal hyperparameters for our model, we used nested cross-validation, also known as double cross-validation (Stone, 1974). The hypertuning process for a single learner is depicted in Figure 1. Each of the five base learners were hypertuned for calculating and comparing the performance metrics of the different models. As can be seen in the Figure, the workflow is as follows: (1) the data are separated into five stratified folds to maintain class balance across folds; (2) one fold is set aside for external evaluation; (3) the four training folds are collectively separated into five stratified folds and used for internally cross-validating each combination of hyperparameter values; (4) the hyperparameters associated with the highest average AUC are then used to fit a model on the four training folds which is then externally evaluated on the fifth fold; and (5) steps 2–4 are repeated with each initial fold being used for testing and the average of each performance metric is taken. Our decision to use AUC for ranking hyperparameters is consistent with previous research (Obuchowski and Bullen, 2018). The hypertuning process is repeated 10 times and the averaged results are reported. The SL works by integrating all five base learners in the training process. It internally evaluates each of the five base learners (steps 1–3 of the hypertuning process) and selects the single best model for external evaluation and further predictions. The VE requires a large number of computations for tuning and each additional base learner increases the computation time exponentially if tuning hyperparameters. Therefore, the VE is not tuned and instead uses the hyperparameters selected for each model when generating the SHAP values (discussed below). It should be noted that this likely artificially inflates the VE performance due to the SHAP hyperparameters being tuned to the entire dataset.

In generating the XAI (LIME and SHAP) values for each learner and task, the hyperparameter combinations are 5-fold cross-validated on the entire dataset and a model is fitted on all the data using the hyperparameters with the highest average AUC.

### 2.4 ML performance metrics

There are multiple performance metrics for evaluating ML classifier performance. These are often used and reported in different combinations and accompanied with varying interpretations. We examined the performance of each classifier using six metrics commonly reported in ML applications. We provisionally separate them for comparison purposes into two clusters: primary and secondary. The first primary metric is AUC, which measures the sensitivity (true positive rate) and specificity (true negative rate) of a model at various decision thresholds. The second primary metric is accuracy, which measures the percentage of all participants being correctly classified. Each of the primary metrics are interpreted with values of 0.5 representing chance, 0.5–0.69 representing poor discrimination, 0.7–0.79 representing acceptable discrimination, 0.8–0.89 representing excellent discrimination, and 0.9–1.0 representing outstanding discrimination (Mandrekar, 2010). The first secondary metric is precision, the percentage of participants correctly identified as the target cohort. Precision is calculated as: $\frac{\text{true}\;\text{positives}}{\text{true}\;\text{positives}+\text{false}\;\text{positives}}$. The second secondary metric is recall (or sensitivity), the percentage of participants from the target cohort that were correctly identified. Recall is calculated as: $\frac{\text{true}\;\text{positives}}{\text{true}\;\text{positives}+\text{false}\;\text{negatives}}$. The third secondary metric is F1 score, a measure of both precision and recall. It is calculated as: $\frac{2*\text{precision}*\text{recall}}{\text{precision}+\text{recall}}$. The fourth secondary metric is Matthew’s Correlation Coefficient (MCC), a single-value summarization of the confusion matrix (a table of: true positives, true negatives, false positives, false negatives) that ranges from −1 (perfect misclassification) to +1 (perfect classification) (Chicco and Jurman, 2020). While MCC is similar to the F1 score, it has the advantage of accounting for dataset imbalances and producing a high score only when the model performs well on the majority of both positive and negative samples. This metric is of particular relevance in the MCI vs. CU task as the MCI cohort has almost double the number of individuals as CU. MCC can be calculated as: $\frac{TP*TN-FP*FN}{\sqrt{\left(TP+FP\right)*\left(TP+FN\right)*\left(TN+FP\right)*\left(TN+FN\right)}}$. In the multi-class dataset (AD vs. MCI vs. SCI vs. CU), precision, recall, F1, and AUC are each calculated by averaging the four one-vs-rest values for each metric. This approach uses the unweighted mean and ignores class imbalance.

### 2.5 Two explainable artificial intelligence algorithms

XAI algorithms complement supervised ML models by providing deeper explanations for why a specific prediction was made and how each feature contributed to the prediction. Notably, although the results of the seven ML algorithms may identify and rank-order the leading predictive features, they typically do not provide comprehensive information regarding interpretation of the observed effects. These XAI algorithms are often model-agnostic, meaning they can be applied to various supervised ML models and the unbiased results can be easily compared with commonly available metrics. We compared two XAI algorithms (i.e., LIME and SHAP) which have considerable promise for neurodegenerative research based on their ability to explain the ML model results. First, we calculated the LIME and SHAP importance values for each feature. This permitted us to determine the features that (1) have the most impact overall in generating predictions for each supervised ML model and (2) are most indicative of a given participant having a specific label. Second, we calculated the composition ratios by dividing the absolute LIME or SHAP value of a single feature by the sum of all absolute feature values and multiplying by 100. These values sum to 100 and give us the percentage that each feature contributes to a prediction as averaged across all samples in a cohort comparison for a single supervised ML model. We applied these XAI algorithms to each supervised ML model except for SL because SL ultimately selects a single base learner from the original five. As a result, computing importance values for SL would be redundant. Also, we do not use the XAI algorithms on the multi-class comparison dataset because the importance values for each feature are binary with respect to each class (positive/negative) and thus we already observe similar values in each of the three pairwise comparison tasks (AD vs. CU, MCI vs. CU, SCI vs. CU). The two XAI algorithms used in this study are described below, followed by a description of the performance and similarity metrics.

#### 2.5.1 Local interpretable model agnostic explanation

LIME is an XAI algorithm that works by sampling around an individual feature vector and fitting a linear function to the predictions that the model generates for the evaluated samples (Ribeiro et al., 2016b). The linear function is used to calculate the magnitude and direction of influence of each input feature independently of the others. LIME has several favorable properties such as being model agnostic (i.e., it can be applied similarly to each of our models) and locally faithful (i.e., closely approximates a model around a single sample). We used the LimeTabularExplainer class with 5,000 samples from the Python library ‘lime’ to generate LIME importance values (Ribeiro, 2016a). To assess an entire model, we averaged the importance values calculated by LIME for each feature over all predictions on the dataset.

#### 2.5.2 SHapley additive exPlanations

SHAP is an XAI algorithm that gives each feature a value indicating its influence on a single prediction by calculating the expected change in the prediction when the feature is introduced (Lundberg and Lee, 2017a). This algorithm is useful for analyzing multiple supervised ML models as it is model-agnostic. In addition, it is an additive feature attribution method like LIME (meaning the explanation model is a linear function), however it does not assume feature independence and uses all combinations of input features in generating the importance values. Calculating SHAP values using this formula requires training a model for each combination of input features and therefore the computation time grows exponentially with the number of features. To address this challenge, we used two types of approximations depending on the ML algorithm. For RF and GB, we used TreeSHAP (Lundberg and Lee, 2017b) which efficiently approximates SHAP values for decision tree methods. For the other algorithms, we used the model agnostic KernelSHAP (Lundberg and Lee, 2017a), which approximates SHAP values using LIME with specific parameters that maintain the properties of SHAP. Both approaches are able to closely approximate SHAP values when features are independent. However, within both approaches the errors increase similarly with feature correlation even though TreeSHAP incorporates dependence between features in its approach and KernelSHAP does not (Aas et al., 2021). The Python library ‘shap’ was used to implement TreeSHAP using the TreeExplainer class and KernelSHAP using the KernelExplainer class (Lundberg, 2018). We evaluated the entire model by averaging SHAP values over all predictions (as was done with LIME).

### 2.6 Comparison metrics for the XAI algorithms

#### 2.6.1 Performance metrics

The XAI performance metrics are calculated for each explanation method independently of the other and are used to directly compare the performance of the two methods. We used five metrics as introduced by Doumard et al. (2023). We identify them as two primary metrics and three secondary metrics for use in comparison. The first primary metric is the mean computation time per instance, which refers to the time it takes to generate the importance values for each sample. Computation time is a relevant metric because if two XAI approaches have similar outputs (which we compare below), the faster of the two is preferred. The second primary metric is robustness, which measures how much the importance value changes for an instance if it is perturbed by a small amount. It is often calculated using maxxj||fxi−fxj||A2xi−xj where f(x_j_) refers to the importance value of x_j_, one of 10 samples from the normal distribution around x_i_: Ɲ (*μ* = x_i_, *σ* = 1e-3). The lower the value, the less the feature importance changes between samples very close to one another and thus the model is more robust. The first secondary metric is the Area Under the cumulative Feature Importance Curve (AUFIC), which measures whether an explainer gives importance to few features or many. It is calculated as $\frac{1}{d}\sum_{i=0}^{d-1}\frac{C_{i}+C_{i+1}}{2}$ where d refers to the total number of features and C_i_ refers to the cumulative importance of the i*th* feature in descending order of importance value. This metric is bounded between 0.5 and 1, with 0.5 meaning equal importance is given to all features and 1 meaning all importance is given to a single feature. The second secondary metric is readability, which measures the correlation between the value of a feature and its influence. It is calculated as $\frac{1}{d}\sum_{i=1}^{d}\;|r\left(x_{i},f\left(x_{i}\right)\right)|$ where r is the Spearman correlation coefficient. A high readability score means the link between a feature and its explanations are more visually obvious in a dependence plot. The third secondary metric is clusterability, which measures the joint contribution of pairs of features. It is calculated as $\frac{2}{d*\left(d-1\right)}\sum_{i,j\in \left[1,\ldots d\right],i\neq j}S(K\left(f\left(x_{i}\right),f\left(x_{j}\right)\right)$ where K is K-Means with 8 clusters (the default number of clusters in scikit-learn’s implementation) and S is the silhouette score. Higher clusterability indicates that the model captures more interactions between features and is thus preferred for a non-linear ML model.

#### 2.6.2 Similarity metrics

The XAI similarity metrics are calculated by directly comparing the importance values and composition ratios generated by LIME and SHAP for each model and dataset and quantify the similarity between the results of both algorithms. We use five metrics to describe the similarity between the two XAI algorithms. The first metric is matching directions—the number of features that share the same direction of influence (i.e., both LIME and SHAP importance values are either positive or negative). The second metric is the top 10 composition overlap—the number of features found in the top 10 features ordered by composition ratio for both algorithms. The third metric is the mean absolute composition difference—the mean of the absolute difference between composition ratios from LIME and SHAP for all features. The smaller this value is, the more similar the composition ratios generated by both algorithms are for each of the features. The fourth metric is the concordance index, a measure of similarity between the lists of features ordered by composition ratios from both LIME and SHAP (calculated as $\frac{\#\text{concordant}\;\text{pairs}}{\#\text{concordant}\;\text{pairs}+\#\text{discordant}\;\text{pairs}}$). A concordance index of 1 means that both lists are ordered exactly the same whereas 0 means that the lists are ordered reverse to one other. The fifth metric is the number of leading predictors in both algorithms. We refer to predictors with a composition ratio greater than 2% as leading predictors. We decided upon this value through initial inspection of SHAP waterfall plots as it appeared to be a natural separating point between a smaller “leading” group of predictors and a larger “following” group.

### 2.7 Leading predictors across XAI-ML combinations

The third RG involves identifying leading predictors and determining their consistency among the 12 combinations of the six ML (excluding SL, as explained in Methods Section 2.5) and two XAI algorithms for each of the AD-related cohorts. We compute both the mean composition ratio across all approaches as well as highlighting features that contributed more than 2% on average to predictions for each combination (i.e., features that have a composition ratio greater than 2.0). We can then calculate the fraction of leading features that the different approaches had in common, as well as focusing on the shared features between the best-performing models for each cohort comparison.

## 3 Results

### 3.1 Supervised ML performance

We report the primary and secondary performance metrics for the seven supervised ML algorithms in each of the four cohort comparison tasks. The metric values are reported in numerical order and comparative interpretations are presented with caution.

#### 3.1.1 Discriminating the AD and CU cohorts

**Figure 2:**
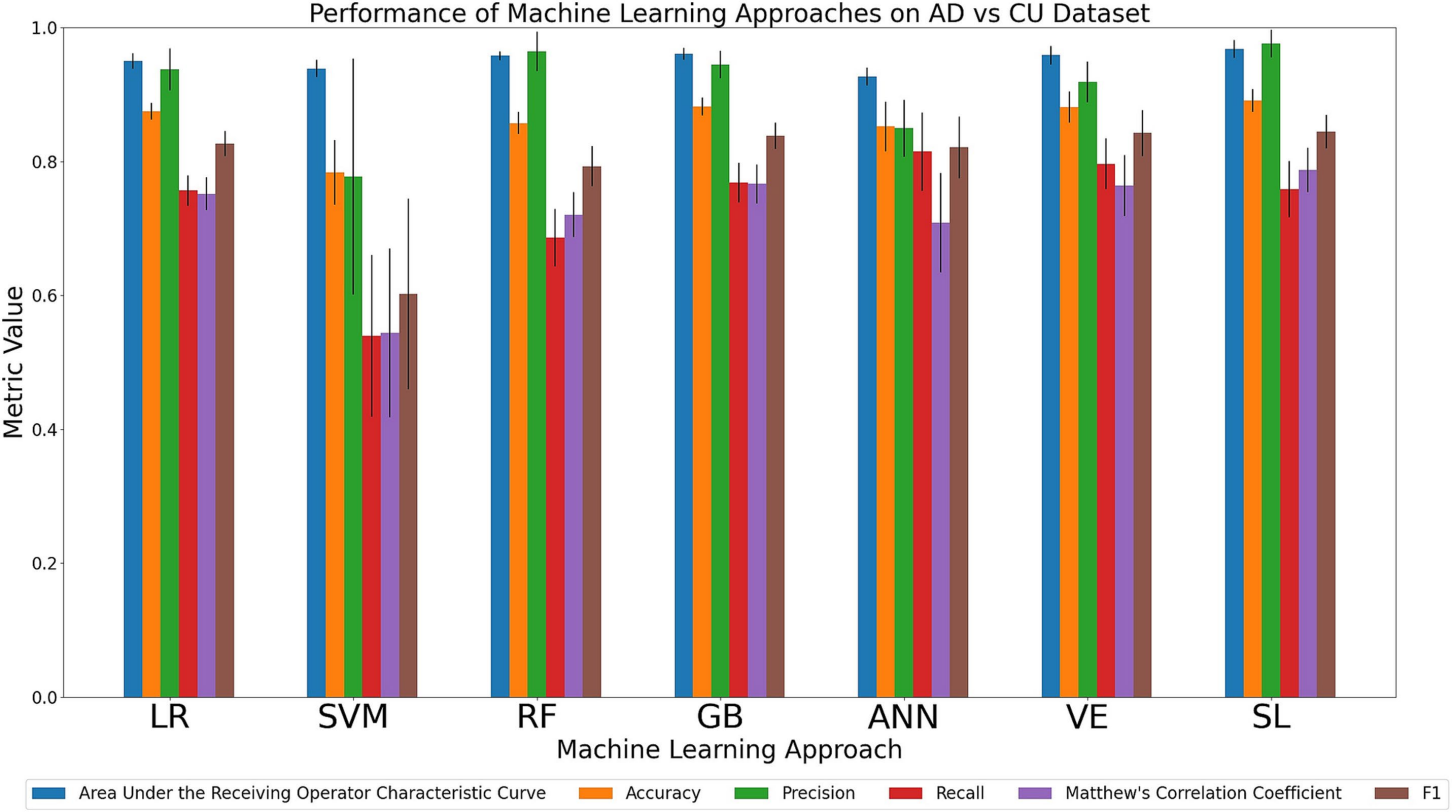
Comparison of mean results of all metrics across ML approaches on the AD vs. CU dataset over 10 trials of 5-fold cross-validation. Error bars represent the standard deviation. Cohort and model acronyms identified in the text.

We compared the relative performance of the seven ML models in discriminating the most clinically extreme cohorts (AD and CU). The results of all six metrics can be viewed in Figure 2 with details in Supplementary Table 7. We first considered the two primary metrics (AUC, accuracy). Overall, we observed that all models displayed outstanding and consistent AUC performance (AUC range: 0.93–0.97) with only minor model differences being observed. A numerically ordered cluster of uniformly high AUC performances included SL (0.97), GB (0.96), VE (0.96), RF (0.96), LR (0.95), SVM (0.94), and ANN (0.93). Accuracy performance varied somewhat more substantially (accuracy range: 0.78–0.89). A numerically ordered cluster of high accuracy performance included SL (0.89), GB (0.88), VE (0.88), LR (0.87), RF (0.86), ANN (0.85), followed by SVM (0.78). As can be seen in Supplementary Table 7, the better performing models (according to the primary metrics) also performed well in the secondary metrics. Specifically, SL achieved the highest precision (0.98; range across all algorithms: 0.78–0.98) and MCC (0.79; range across all algorithms: 0.54–0.79). SL also tied with GB for highest F1 (0.84; range across all algorithms: 0.60–0.84), whereas ANN achieved the highest recall (0.81; range across all algorithms: 0.54–0.81). Collectively, these results indicate that most models performed well in the task of discriminating between two most clinically extreme cohorts. In this context, SL consistently performed notably well in the task of discriminating between AD and CU, achieving the highest AUC, accuracy, precision, and MCC. GB and ANN also performed consistently well in secondary metrics. The success of SL on this dataset highlights the importance of tuning a model whereas the success of GB and ANN suggests that nonlinear methods are well-suited for this dataset.

#### 3.1.2 Discriminating the MCI and CU cohorts

**Figure 3:**
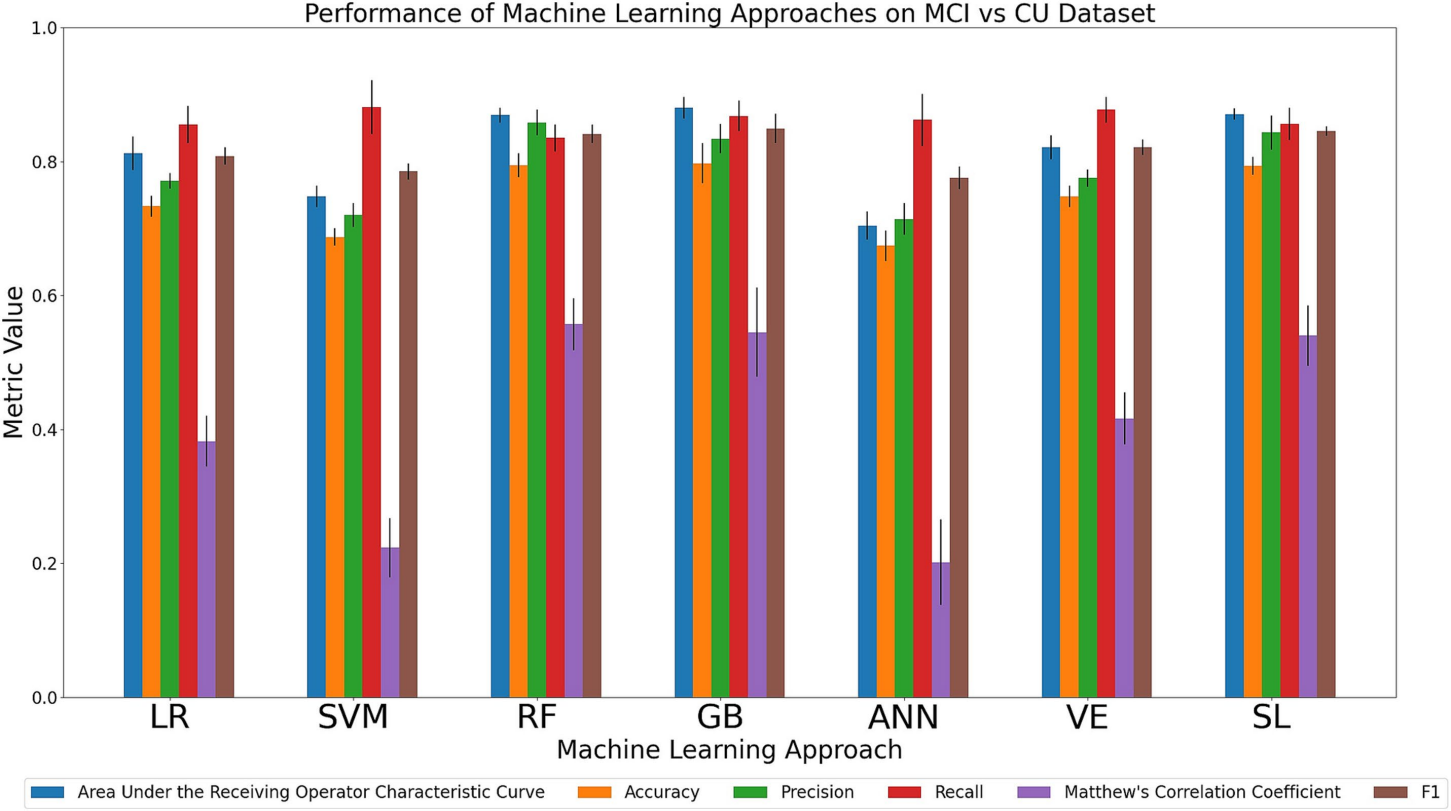
Comparison of mean results of all metrics across ML approaches on the MCI vs. CU dataset over 10 trials of 5-fold cross-validation. Error bars represent the standard deviation. Cohort and model acronyms identified in the text.

We compared the relative performance of the seven ML models in discriminating between the MCI and CU cohort. The results of all six metrics can be seen in Figure 3 and Supplementary Table 8. We first considered the primary metrics (AUC, accuracy). Overall, the models displayed varying AUC scores of either excellent or acceptable performance (AUC range: 0.70–0.88). Regarding accuracy, the models displayed either acceptable or poor discrimination performance (accuracy range: 0.67–0.80). For AUC, GB (0.88), SL (0.87) and RF (0.87) achieved the highest values, followed numerically by VE (0.82), LR (0.81), SVM (0.75), and ANN (0.70). For accuracy, GB (0.80), RF (0.79), and SL (0.79) were characterized by the best performances, followed numerically by VE (0.74), LR (0.73), SVM (0.69), and ANN (0.67). Regarding the secondary metrics: RF had the highest precision (0.86; range across all algorithms: 0.71–0.86) and MCC (0.56; range across all algorithms: 0.20–0.56); SVM and VE had the highest recall (0.88; range across all algorithms: 0.84–0.88); GB and SL had the highest F1 score (0.85; range across all algorithms: 0.77–0.85). In sum, our comparison indicates that GB was consistently high performing in the task of discriminating between MCI and CU in both primary metrics as well as one of the secondary metrics (F1 score). RF was notably the highest in two of the secondary metrics (precision, MCC). Other models (VE, SVM, and SL) scored the highest in one of the secondary metrics. The success of GB further emphasizes the benefit of using decision-tree-based methods on categorical data.

#### 3.1.3 Discriminating the SCI and CU cohorts

**Figure 4:**
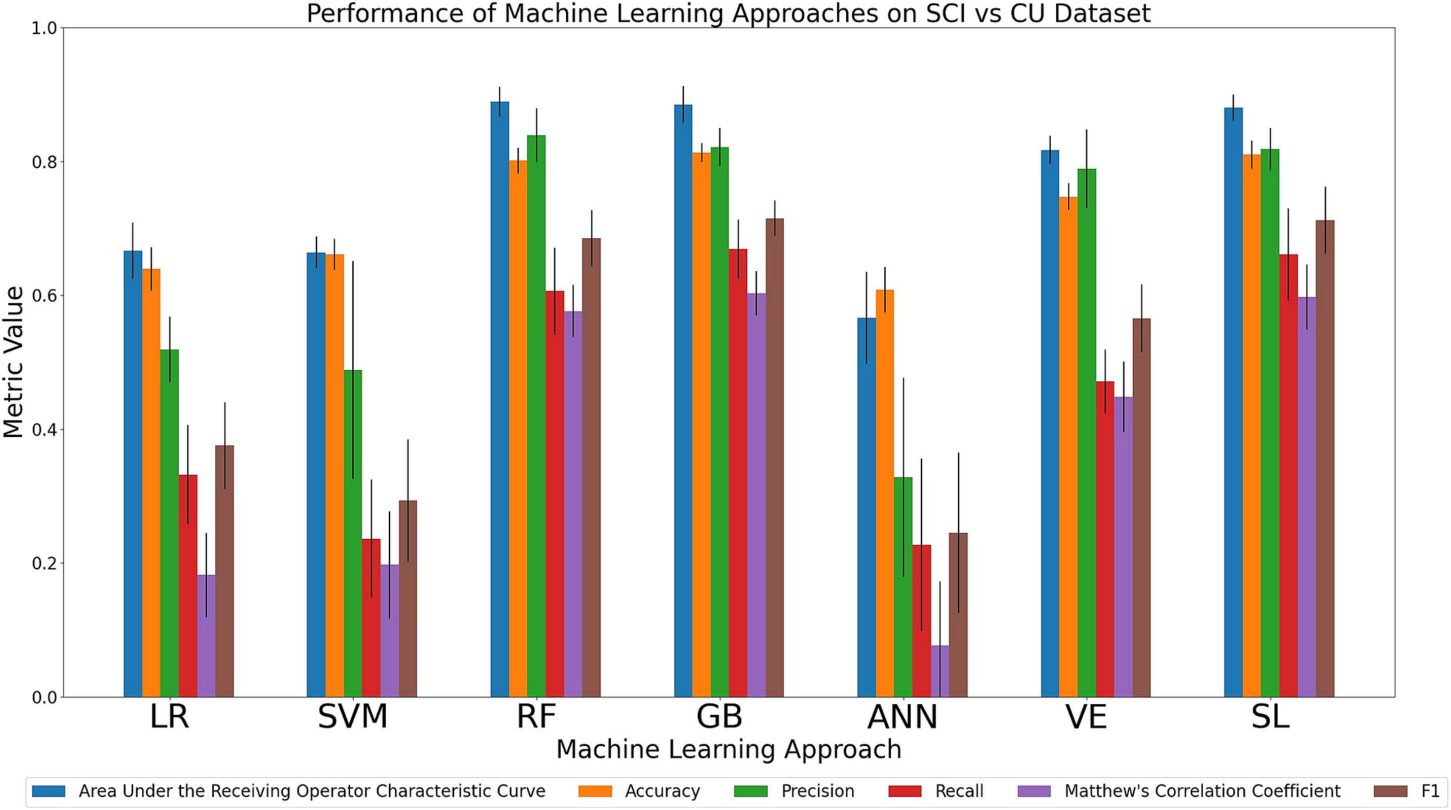
Comparison of mean results of all metrics across ML approaches on the SCI vs. CU dataset over 10 trials of 5-fold cross-validation. Error bars represent the standard deviation. Cohort and model acronyms identified in the text.

We compared the relative performance of the seven ML models in discriminating between the SCI and CU cohort. The results of all six metrics can be seen in Figure 4 and Supplementary Table 9. Performance of the models in the primary metrics (AUC, accuracy) varied notably, but consistently. The observed AUC and accuracy values ranged from poor to excellent (AUC range: 0.57–0.89; accuracy range: 0.61–0.81). For AUC, RF (0.89), GB (0.89) and SL (0.88) achieved the highest scores, followed numerically by VE (0.82), LR (0.67), SVM (0.66), and ANN (0.57). For accuracy, GB (0.81), SL (0.81), and RF (0.80) achieved the highest accuracy scores, followed numerically by VE (0.75), SVM (0.66), LR (0.64), and ANN (0.61). As can be seen in the Figure, the models that achieved the highest primary metrics also achieved the highest secondary metrics. Regarding the secondary metrics, RF had the highest precision (0.84; range across all algorithms: 0.33–0.84) whereas GB had the highest recall (0.67; range across all algorithms: 0.23–0.67), F1 score (0.72; range across all algorithms: 0.25–0.72), and tied with SL for the highest MCC (0.60; range across all algorithms: 0.08–0.60). In sum, our comparison indicates that SL performed consistently well, however, RF and GB best discriminated between SCI and CU. Specifically, RF achieved the highest AUC and precision and GB achieved the highest accuracy, recall, and F1 score. As noted in the previous two comparison tasks, the decision-tree-based methods performed well on the categorical data.

#### 3.1.4 Simultaneous discrimination of AD, MCI, SCI, and CU cohorts

**Figure 5:**
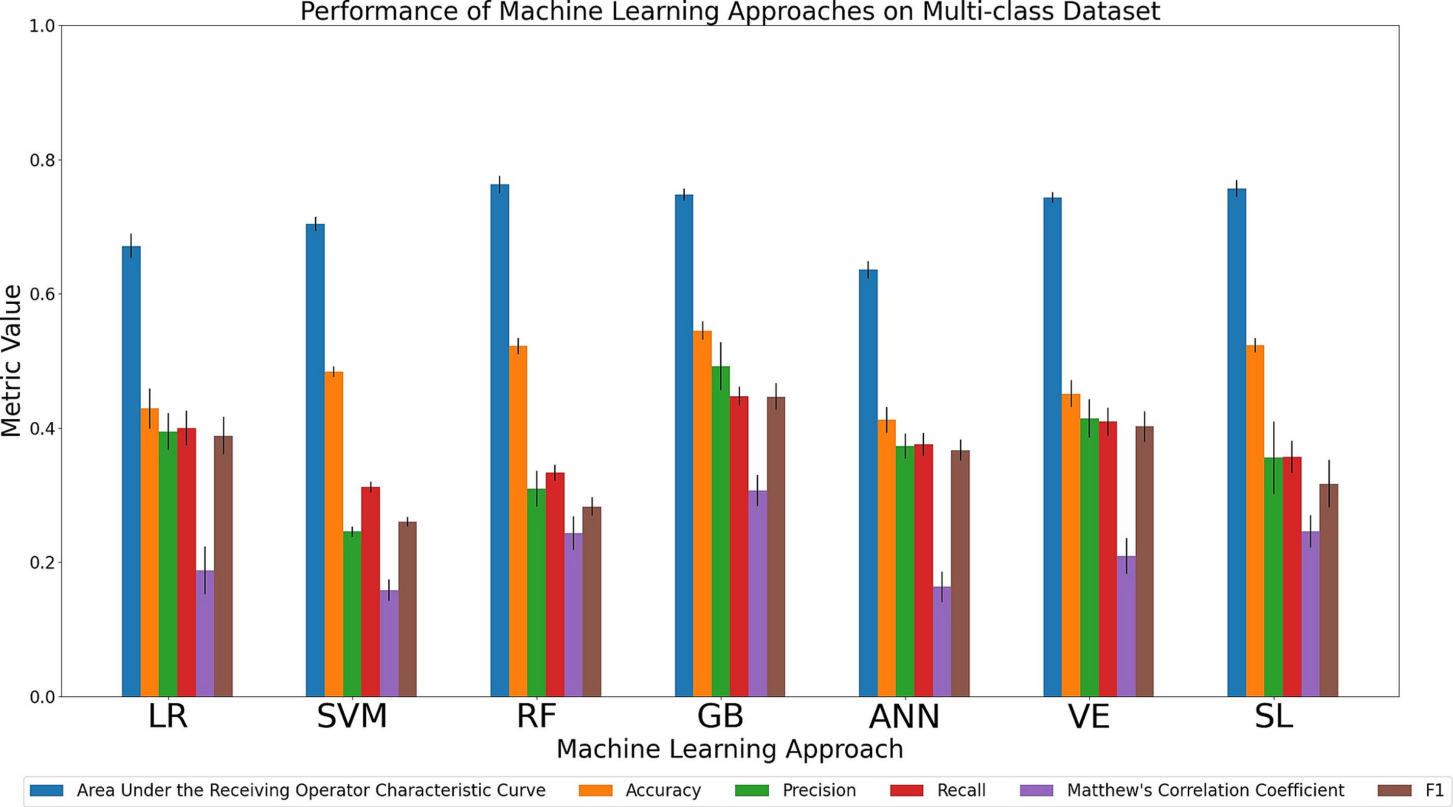
Comparison of mean results of all metrics across ML approaches on the AD vs. MCI vs. SCI vs. CU dataset over 10 trials of 5-fold cross-validation. Error bars represent the standard deviation. Cohort and model acronyms identified in the text.

Discrimination tasks involving multiple cohorts is a clinically relevant but computationally challenging task for ML models. Specifically, the models must learn substantially more parameters due to the increase in the number of possible classifications for a given instance, of which only one is correct. In this complex discrimination task, a model choosing classes at random would have an average accuracy of 0.25 compared to the corresponding average accuracy of 0.5 that would constitute random classification in a binary task. We compared the relative performance of the seven ML models in simultaneously discriminating between all four cohorts (AD, MCI, SCI, and CU). The results of all six metrics can be seen in Figure 5 and Supplementary Table 10. Considering the primary metrics (AUC, accuracy), the models displayed similar results, achieving either poor or acceptable AUC performance (AUC range: 0.64–0.76) and below-poor or poor accuracy performance (accuracy range: 0.41–0.55). For AUC, RF and SL both had the highest AUC (0.76) followed closely numerically by GB (0.75) and VE (0.74), and then SVM (0.70), LR (0.67), and ANN (0.64). For accuracy, GB was characterized by the relatively best performance (0.55) followed closely numerically by SL (0.52) and RF (0.52), with SVM (0.48), VE (0.45), LR (0.43), and ANN (0.41) performing at a poorer level. Considering the secondary metrics, GB achieved the highest precision (0.49; range across all algorithms: 0.25–0.49), recall (0.45; range across all algorithms: 0.31–0.45), MCC (0.31; range across all algorithms: 0.16–0.31), and F1 (0.45; range across all algorithms: 0.26–0.45). In sum, our comparison indicates that GB outperformed the other models in the task of simultaneously discriminating between the four cohorts in achieving the highest scores in accuracy and all secondary metrics while RF and SL scored the highest AUC.

Across all tasks we observed that RF, GB, and SL were consistently ranked high, with GB ranking with the highest performance on the largest number of metrics.

### 3.2 XAI performance comparisons

Following the performance results for all seven ML models, as computed for each of the four discrimination tasks, we computed follow-up LIME and SHAP values for six of the models (excluding SL, as explained in Methods Section 2.5) within each of the three pairwise cohort discrimination tasks. For SHAP, we used the decision-tree-specific version TreeSHAP for RF and GB, and the more general KernelSHAP for LR, SVM, ANN, and VE. The hyperparameters used in these tasks are reported in Supplementary Tables 3–6.

#### 3.2.1 Discriminating the AD and CU cohorts

The XAI performance results for the AD vs. CU discrimination task are reported in Table 3. We first considered the primary metrics (mean computation time, robustness). Computation time refers to the time it takes to compute the importance values for a single instance. Results indicated that LIME performed faster than KernelSHAP and slower than TreeSHAP. LIME had an average mean computation time of 0.09 s across non-decision-tree models, whereas KernelSHAP had an average of 19.94 s. When applied to RF and GB, LIME had an average time of 0.07 s and TreeSHAP had an average time less than 0.01 s. Robustness refers to how much the importance values change when the input features are perturbed by a small amount, with a lower value meaning the XAI algorithm is more robust. For this metric, SHAP had a lower value than LIME with every model. We then considered the secondary metrics (AUFIC, readability, clusterability). We observed that the AUFIC values (which reflect how the weight is distributed across features with 0.5 meaning all features are equally important and 1 meaning only a single feature is important) were similar between LIME and SHAP for each model. Across all models, LIME had an average AUFIC of 0.77 whereas SHAP had an average of 0.75, indicating that both approaches distribute importance values over many features rather than highlighting a single feature. SHAP achieved a higher readability value than LIME for every model indicating that the links between feature values and their importance values are visually clearer in SHAP dependence plots. LIME and SHAP had similar clusterability scores (which refer to the degree that an approach captures interactions between features, with lower values meaning fewer interactions are represented) with averages of 0.44 and 0.43, respectively.

**Table 3:** Intra-explainer comparison of LIME and SHAP for each algorithm on the AD vs CU dataset.

Model	Mean Computation Time (seconds) [0, ∞] ꜜ		Robustness [0, ∞] ꜜ		AUFIC [0.5,1]		Readability [0,1] ꜜ		Clusterability [−1,1] ꜜ	
	LIME	SHAP	LIME	SHAP	LIME	SHAP	LIME	SHAP	LIME	SHAP
LR	**0.04**	15.14	79.49	**65.88**	0.75	**0.75**	0.08	**0.57**	0.40	**0.41**
SVM	**0.11**	17.58	271.24	**92.26**	**0.75**	0.70	0.09	**0.49**	**0.44**	0.37
RF	0.08	**<0.01**	96.46	**2.53**	**0.83**	0.82	0.07	**0.55**	0.45	**0.48**
GB	0.06	**<0.01**	221.33	**75.82**	**0.83**	0.80	0.08	**0.51**	0.47	**0.48**
ANN	**0.04**	20.80	468.08	**63.00**	0.71	**0.73**	0.07	**0.63**	**0.43**	0.42
VE	**0.18**	26.24	213.02	**76.29**	**0.74**	0.72	0.08	**0.56**	**0.43**	0.39
Avg.	**0.09**	13.29	224.94	**63.24**	**0.77**	0.75	0.08	**0.55**	**0.44**	0.43

The ranges of the metrics are displayed beneath the metric name for each column. ꜛ indicates that higher values on the corresponding metric denote better performance. ꜜ indicates that lower values on the corresponding metric denote better performance. Values in bold print identify the best performance by a model for each metric. AD, Alzheimer’s Disease; CU, Cognitively Unimpaired; AUFIC, Area Under the cumulative Feature Importance Curve; LIME, Local Interpretable Model agnostic Explanation; SHAP, SHapley Additive exPlanations; LR, Logistic Regression; SVM, Support Vector Machine; RF, Random Forest; GB, Gradient-Boosted trees; ANN, Artificial Neural Network; VE, Voting Ensemble. Mean computation time and robustness are considered primary metrics, the rest are considered secondary.

#### 3.2.2 Discriminating the MCI and CU cohorts

The XAI performance results for the MCI vs. CU discrimination task are reported in Table 4. We first considered the primary metrics (mean computation time, robustness). For mean computation time, LIME had faster times in all models except RF and GB. LIME had an average mean computation of 0.16 s across non-decision-tree models whereas KernelSHAP had an average of 32.07 s. For RF and GB, LIME had an average mean computation time of 0.20 s and TreeSHAP had an average of less than 0.01 s. For robustness, SHAP had a lower score than LIME in all models indicating that it is more robust. Considering secondary metrics (AUFIC, readability, clusterability), both LIME and SHAP had average AUFIC scores of 0.79 across all models. SHAP had much higher readability and scores than LIME for all ML models. Although SHAP also achieved higher clusterability scores than LIME across all ML models, the differences were small (*Mean* = 0.06).

**Table 4:** Intra-explainer comparison of LIME and SHAP for each algorithm on the MCI vs CU dataset.

Model	Mean Computation Time (seconds) [0, ∞] ꜜ		Robustness [0, ∞] ꜜ		AUFIC [0.5, 1]		Readability [0, 1] ꜜ		Clusterability [−1, 1] ꜜ	
	LIME	SHAP	LIME	SHAP	LIME	SHAP	LIME	SHAP	LIME	SHAP
LR	**0.08**	24.55	178.81	**47.87**	**0.90**	0.87	0.07	**0.33**	0.47	**0.56**
SVM	**0.14**	26.39	151.62	**57.11**	0.74	**0.74**	0.09	**0.66**	0.40	**0.47**
RF	0.29	**<0.01**	63.70	**2.57**	**0.81**	0.79	0.05	**0.51**	0.44	**0.46**
GB	0.10	**<0.01**	96.84	**2.76**	0.84	**0.85**	0.05	**0.55**	0.46	**0.50**
ANN	**0.08**	33.21	255.27	**71.02**	0.67	**0.69**	0.07	**0.71**	0.41	**0.48**
VE	**0.36**	44.13	149.21	**51.93**	**0.78**	0.77	0.07	**0.61**	0.42	**0.47**
Avg.	**0.18**	21.38	149.24	**38.88**	**0.79**	0.79	0.07	**0.56**	0.43	**0.49**

The ranges of the metrics are displayed beneath the metric name for each column. ꜛ indicates that higher values on the corresponding metric denote better performance. ꜜ indicates that lower values on the corresponding metric denote better performance. Values in bold print identify the best performance by a model for each metric. MCI, Mild Cognitive Impairment; CU, Cognitively Unimpaired; AUFIC, Area Under the cumulative Feature Importance Curve; LIME, Local Interpretable Model agnostic Explanation; SHAP, SHapley Additive exPlanations; LR, Logistic Regression; SVM, Support Vector Machine; RF, Random Forest; GB, Gradient-Boosted trees; ANN, Artificial Neural Network; VE, Voting Ensemble. Mean computation time and robustness are considered primary metrics, the rest are considered secondary.

#### 3.2.3 Discriminating the SCI and CU cohorts

The XAI performance results for the SCI vs. CU discrimination task are reported in Table 5. Considering the primary metrics (mean computation time, robustness), LIME had faster mean computation times for all models except RF and GB. The average mean computation time across the KernelSHAP approaches was 0.08 s for LIME and 6.78 s for KernelSHAP. Across the TreeSHAP approaches, the average time was 0.07 s for LIME and less than 0.01 s for TreeSHAP. SHAP had lower robustness scores than LIME across all models indicating it is more robust. Considering the secondary metrics (AUFIC, readability, clusterability), LIME and SHAP both had similar AUFIC scores (*Mean* = 0.77 and 0.73, respectively) and clusterability scores (*Mean* = 0.43 and 0.47, respectively). For the readability metric, SHAP had higher scores than LIME for all models.

**Table 5:** Intra-explainer comparison of LIME and SHAP for each algorithm on the SCI vs CU dataset.

Model	Mean Computation Time (seconds) [0, ∞] ꜜ		Robustness [0, ∞] ꜜ		AUFIC [0.5,1]		Readability [0,1] ꜜ		Clusterability [−1,1] ꜜ	
	LIME	SHAP	LIME	SHAP	LIME	SHAP	LIME	SHAP	LIME	SHAP
LR	**0.04**	4.54	66.73	**42.30**	0.72	**0.73**	0.11	**0.65**	0.39	**0.52**
SVM	**0.05**	5.66	209.45	**66.91**	**0.74**	0.71	0.09	**0.58**	0.40	**0.47**
RF	0.08	**<0.01**	68.14	**3.65**	**0.83**	0.75	0.07	**0.49**	**0.45**	0.43
GB	0.09	**<0.01**	187.35	**96.14**	**0.85**	0.79	0.07	**0.51**	0.47	**0.49**
ANN	**0.05**	5.67	85.57	**55.08**	**0.74**	0.70	0.09	**0.65**	0.41	**0.51**
VE	**0.18**	11.23	126.55	**91.34**	**0.76**	0.72	0.08	**0.45**	**0.44**	0.38
Avg.	**0.08**	4.52	123.97	**59.40**	**0.77**	0.73	0.09	**0.55**	0.43	**0.47**

The ranges of the metrics are displayed beneath the metric name for each column. ꜛ indicates that higher values on the corresponding metric denote better performance. ꜜ indicates that lower values on the corresponding metric denote better performance. Values in bold print identify the best performance by a model for each metric. SCI, Subjective Cognitive Impairment; CU, Cognitively Unimpaired; AUFIC, Area Under the cumulative Feature Importance Curve; LIME, Local Interpretable Model agnostic Explanation; SHAP, SHapley Additive exPlanations; LR, Logistic Regression; SVM, Support Vector Machine; RF, Random Forest; GB, Gradient-Boosted trees; ANN, Artificial Neural Network; VE, Voting Ensemble. Mean computation time and robustness are considered primary metrics, the rest are considered secondary.

#### 3.2.4 Summary

Across all three tasks, LIME had consistently faster mean computation times than SHAP on models when it was using KernelSHAP (LR, SVM, ANN, VE) and slower times than SHAP on models when it was using TreeSHAP (RF and GB). SHAP achieved higher robustness and readability scores than LIME across all ML models. Both XAI techniques displayed similar AUFIC and clusterability values to one another across each task and ML model. These results favor SHAP over LIME, particularly when applying TreeSHAP to the decision-tree methods RF and GB, which performed consistently well in the discrimination tasks. SHAP’s faster computation time with TreeSHAP, coupled with its higher robustness and readability, means that results are more consistent and interpretable compared to LIME.

### 3.3 XAI similarity of results comparisons

After comparing the performance of both LIME and SHAP individually, we compare metrics that quantify the similarity in the results between both XAI techniques across the ML models. These include the five previously described metrics to estimate similarity: (1) the number of features with matching directions of influence (ranging from 0 to the number of features in the dataset), (2) number of overlapping features between the top 10 features by composition for each XAI approach (ranging from 0 to 10), (3) the mean absolute composition difference between features (ranging from 0 to 100), (4) the concordance index between the lists of features ranked by composition ratio for each approach (ranging from 0 to 1), and (5) the number of leading predictors in each approach [ranging from 0 to 50 (since each leading feature must have more than 2% composition ratio)].

#### 3.3.1 Discriminating the AD and CU cohorts

The XAI similarity results for the AD vs. CU discrimination task are found in Supplementary Table 11. LIME and SHAP displayed high overlap between the top 10 features ranked by composition ratios (range: 8–9) and high agreement between all features ranked by composition ratio, as displayed by the high concordance indexes (range: 0.77–0.91). Furthermore, for all model comparisons, the two XAI algorithms had little mean differences between the absolute composition values across features (range 0.27–0.55), on average differing by less than 1%. The number of leading predictors identified was also similar for both LIME and SHAP across all ML models except for SVM, where the number of leading predictors for LIME was double that of SHAP (10 vs. 5). Where the two approaches differ however, is the direction of influence (i.e., whether the feature contributes to a positive or negative classification). Regarding the number of features with matching directions of influence, LIME and SHAP differed in their predictions with an average across all ML models of 36/74 (49%) and the highest number of matching feature directions being 44/74 (59%) as seen in the ANN comparison. Overall, LR displayed the most similarity between LIME and SHAP according to difference in mean absolute composition ratios and concordance index. This result is unsurprising since LR is a linear approach and thus there are no feature interactions that SHAP can take advantage of and outperform LIME.

#### 3.3.2 Discriminating the MCI and CU cohorts

The XAI similarity results for the MCI vs. CU discrimination task are reported in Supplementary Table 12. The XAI algorithms both agreed in terms of composition ratios with high overlap both in the top 10 features (range: 7–9), concordance indexes (range: 0.76–0.92), and low mean composition differences (range: 0.24–0.58). As for the number of leading predictors, LIME and SHAP both had similar results with the largest difference being observed in the case of ANN with LIME having 18 leading predictors and SHAP having 12. Considering the number of features with matching directions of influence between approaches, LIME and SHAP only agree on half the features with an average number of matching directions of 31.83 (49%) and the highest being 35 (54%) from VE. In this task, the ML model with the most similar results between LIME and SHAP was SVM, which is also a linear approach and thus it makes sense that both XAI techniques would give similar outputs.

#### 3.3.3 Discriminating the SCI and CU cohorts

The XAI similarity results for the SCI vs. CU discrimination task are reported in Supplementary Table 13. In this task, LIME and SHAP show less similarity in composition ratios than in the other comparison tasks. The top 10 composition overlap has a much larger range of 1–9 with ANN only sharing a single feature across both XAI feature rankings. The mean absolute composition difference and concordance indexes also have larger ranges of 0.31–1.43 and 0.48–0.92, respectively. The number of leading predictors between LIME and SHAP differed by at least two across ML models with a maximum difference of seven observed in the case of GB (5 vs. 12). Much like the previous two tasks, LIME and SHAP only agree on the direction of influence of around half the features with an average number of matching directions of 30.50 (48%) and the highest being 34 (53%) from SVM. As observed in the previous tasks, LR and SVM generally had the highest similarities between XAI approaches indicating that LIME and SHAP perform similarly on linear approaches.

#### 3.3.4 Summary

Across all three tasks, we observed that LIME and SHAP had similar composition ratios as displayed by the generally high top 10 composition overlap and concordance indexes and low mean absolute composition difference scores. However, the XAI techniques both differed substantially in terms of the directions of influence across features, with agreement occurring for about half of the feature directions. This means that although LIME and SHAP agree on how important each feature is, they sometimes disagree on how that feature impacts predictions. The results between the two XAI algorithms were similar in both the AD vs. CU and MCI vs. CU tasks, but similarity dropped off in the SCI vs. CU, likely due to the ML model performance drop off observed earlier. We also noticed that the most similar outputs between LIME and SHAP were typically observed when applied to LR and SVM. This can likely be explained by LR and SVM being linear ML algorithms, meaning that SHAP cannot utilize any feature interactions to provide more informed importance values.

### 3.4 Leading predictors across ML-XAI combinations

We compared the leading predictors (i.e., predictors with >2% composition ratio) across pairs of supervised ML models and XAI algorithms for each cohort comparison task. Specifically, we report and compare the leading features as determined by each of LIME and SHAP for the top performing models (according to the primary metrics) in each dataset.

#### 3.4.1 Discriminating the AD and CU cohorts

The total list of features (*N* = 75) and leading predictors for the AD vs. CU task can be found in Supplementary Table 14. Across all combinations of the six ML and two XAI approaches we observed six leading predictors that appeared in at least 10 of the 12 ML-XAI combinations. These predictors and their mean composition ratios were olfaction (9.77), sex (8.64), memory QOL (8.20), handling money (3.81), grip strength (2.98), and timed walk (2.98). We observed that 27 (36%) of the total predictors were identified as a leading predictor by at least one ML-XAI combination. The two best performing base ML models, RF and GB, shared 11 out of the 15 leading predictors identified by either approach when using LIME (14 identified in total by RF, 12 by GB), and 13/15 when using SHAP (14 identified by RF, 14 by GB).

#### 3.4.2 Discriminating the MCI and CU cohorts

The total list of features (*N* = 65) and leading predictors for the MCI vs. CU task can be found in Supplementary Table 15. The leading predictors observed in at least 10 of the 12 combinations were memory QOL (14.20), sex (9.20), grip strength (5.48), pulse pressure (4.56), and self-rated health (3.03). We observed that 30 (46%) of the total predictors were identified as a leading predictor by at least one pair of ML and XAI approaches. RF and GB shared 8/14 leading predictors when using LIME (13 identified in total by RF, 9 by GB), and 7/14 when using SHAP (9 identified by RF, 12 by GB).

#### 3.4.3 Discriminating the SCI and CU cohorts

The total list of features (*N* = 64) and leading predictors for the SCI vs. CU task can be found in Supplementary Table 16. Although no leading predictors were observed across all 12 ML-XAI combinations, memory QOL (9.77) and lymphocytes number (8.13) were the leading predictors in 11/12 and 10/12 of the combinations. We observed that 45 of the total predictors were identified as a leading predictor by at least one combination. RF and GB, the best two performing models, shared 5/11 leading features when using LIME (11 identified in total by RF, 5 by GB) and 6/15 leading features when using SHAP (9 identified by RF, 12 by GB).

#### 3.4.4 Summary

Overall, we observed considerable consistency in the identification of leading predictors across the ML-XAI combinations. We identified several unanimous leading predictors in the AD vs. CU and MCI vs. CU tasks and several with high overlap in the SCI vs. CU task. We also observed that as the clinical gap between cohorts widened, the disagreement between models increased meaning the total number of unique leading predictors across approaches also increased. The results of our experiments reiterate the goal of these XAI algorithms: to estimate how a ML model uses features to make predictions. Therefore, the importance values are affected not only by the type of ML algorithm applied to the dataset, but also by the corresponding performance metrics. Consequently, the results of XAI algorithms are more reliable when the models perform better, particularly when they achieve higher accuracy. This explains the higher variance in leading predictors we observed within the clinically similar cohort comparison task of SCI vs. CU. Additionally, depending on the dataset, there may be different combinations of features that lead to the same prediction. Although the results of the XAI algorithms may not necessarily point to a direct causal link between the leading predictive feature and clinical cohort classifications, they are useful for choosing features in the context of building ML predictors or designing neurodegenerative datasets and studies.

## 4 Discussion

As an AI-derived data-driven analytic technique, ML has been increasingly used in aging and neurodegenerative disease research. Such research has addressed not only the (1) differential diagnosis of neurodegenerative diseases, but also (2) identification of leading disease biomarkers, risk and protective factors that discriminate among early clinical conditions, (3) detection and characterization of risk-related and etiological subgroups, and (4) ascertainment of potential modifiable targets for precision intervention protocols (Drouin et al., 2022; Iturria-Medina et al., 2016; McFall et al., 2023). The dramatic growth of ML technology has produced important advances in applications, including the variety and capacity of supervised learning and XAI algorithms available to researchers. The present study focused purely on the use of ML and XAI in neurodegenerative research rather than in a clinical setting and developed a comprehensive set of criteria for evaluating ML and XAI performance in a typical multi-cohort study of aging and neurodegeneration. Accordingly, we addressed several crucial issues facing researchers as they select and deploy an ML research procedure. The challenges for researchers include: (1) which algorithms to choose, (2) whether to use one or a combination of algorithms, (3) how to evaluate and compare a candidate set of algorithms, and (4) how to incorporate and compare XAI algorithms for post-hoc interpretation.

Our objective was to systematically compare prominent supervised ML and XAI algorithms in specific metrics of performance with discrete multi-feature datasets across formally classified cohorts within the AD spectrum. Our specific goals were to (1) examine computational performance across seven common and promising supervised ML algorithms for neurodegeneration research, (2) investigate the relative performance and similarity between two commonly used post-hoc XAI algorithms (SHAP, LIME), and (3) informally compare the similarity between leading predictors determined by the XAI algorithms when applied to the ML models used in each of the pairwise comparison tasks. Our results provide useful guidelines for the expanding number of researchers applying AI-derived ML technologies to a variety of aging neuroscience datasets.

For the first goal, we compared the performances of the seven supervised ML algorithms (LR, SVM, RF, GB, ANN, VE, SL) according to two primary (AUC, accuracy) and four secondary (precision, recall, MCC, F1) metrics on three binary and one multi-class discrimination tasks involving AD-related cohorts (AD, MCI, SCI, CU). Although other studies have assessed various ML algorithms for classification tasks using aging and neurodegeneration datasets, our study provides uniquely comprehensive information. We systematically compared (1) all selected algorithms using the same multi-feature dataset (Fathi et al., 2022; Pellegrini et al., 2018), (2) a substantial number of ML and XAI algorithms (James et al., 2021; Zhu et al., 2020), (3) a wide selection of differentiated important ML performance metrics (Joshi et al., 2010), and (4) pairwise classification performance among the well-characterized AD-related cohorts. A key finding was that, across algorithms, model performance decreased as the positive clinical class extended further away from benchmark CU cohort (i.e., AD performance > MCI performance > SCI performance) and dropped off significantly in the multi-class classification task. This pattern suggests that ML algorithms are more effective at distinguishing between cohorts with significant differences, rather than those that are quite similar. Moreover, when analyzing the results across the three binary tasks, we observed that the overall performance of certain ML models declined more significantly than others, illustrating that the differences become more pronounced as the tasks increase in difficulty. Specifically, we observed that two base models (RF, GB) and one ensemble model (SL) performed consistently well across all tasks, most notably outperforming the other base models in the demanding comparison of clinically neighboring cohorts (SCI vs. CU). This suggests that nonlinear models, particularly decision-tree, are best-suited for the task of discriminating between clinical neurodegeneration cohorts using discrete multi-feature data.

When comparing model performance across the three binary discrimination tasks, we observed both consistent and unique trends within each comparison. In the AD vs. CU task, a discrimination of the two cohorts most clinically and pathologically different, we observed two notable results. First, all models achieved similarly outstanding AUC performance; however, SVM achieved a slightly lower accuracy than the other models which were clustered together. This result is interesting because LR and SVM are both linear methods but only SVM underperformed compared to the other models. We also observed that SL achieved the highest scores in three of the four secondary metrics (precision, MCC, and F1 score). Notably, SL is an ensemble algorithm that internally validates base learners and uses the best performing one for final predictions. Since all models achieved similar scores across the various metrics, it is unclear which base model is most likely to be selected by SL. In the MCI vs. CU task, we observed two results different from the preceding task. First, we observed that, in terms of the primary metrics, RF, GB, and SL performed similarly well (although lower than that of the AD vs. CU task) and exceeded the other models. Second, we observed more variation in the secondary metrics. Specifically, we found that: (1) RF was characterized by the highest precision and MCC; (2) SVM and VE were characterized by the highest recall; and (3) GB and SL were characterized by the highest F1 score. In the SCI vs. CU task, a discrimination of two clinically neighboring and similar cohorts, we observed similar trends of the primary metrics as seen in the previous task but different secondary metric results. We again observed RF, GB, and SL performed similarly to one another and outperformed the other models. This trend indicates that SL is likely using RF and GB to make predictions in these more difficult tasks. We also observed these three models outperforming the others according to the secondary metrics with RF achieving the highest precision whereas GB achieved the highest recall, F1 score, and MCC (tying with SL). In the four-way classification task, we observed a significant drop-off in performance across all metrics which is to be expected since the total number of classes is double that of the binary tasks. Similar to the previous two tasks, RF, GB, and SL achieved the highest primary metrics scores but were more closely followed by the other models. We also observed that GB achieved the highest scores in all four secondary metrics. The consistently high performance of RF and GB as well as the poor performance of ANN (the only other non-linear base model) indicate that decision-tree-based methods perform well on discrete data for classification tasks, which is in line with other studies (Climent et al., 2018; Gray et al., 2013; Moore et al., 2019; Vyas et al., 2022). The detectable performance difference between RF/GB and ANN is likely due to ANNs requiring many samples to generalize well and perform optimally, especially when hypertuning (Ying, 2019); we acknowledge that the present dataset contains a relatively small number of samples. In sum, the results suggest that RF and GB are best suited for datasets with common discrete neurodegeneration variables (e.g., age, sex, Mini-Mental State Examination (MMSE), Apolipoprotein E (APOE) gene) (Bennett et al., 2018; Chertkow et al., 2019; Morris et al., 2006; Petersen et al., 2010a). Therefore, we suggest using either of the decision-tree approaches RF or GB. For likely improved performance, it is recommended to incorporate both algorithms into the ensemble method SL alongside other promising algorithms.

Subsequently, we applied two common post-hoc XAI algorithms (LIME and SHAP) to the ML models and compared their relative performance and similarity. While studies have been conducted to compare the performances of LIME and SHAP (Amparore et al., 2021; Doumard et al., 2023; ElShawi et al., 2020), few have assessed the similarities between the importance values generated by each algorithm. Our work assesses both performance and similarities of the two approaches using the same dataset. We first compared the two algorithms according to independent performance metrics. These metrics were derived from a study by Doumard et al. (2023) in which they compared LIME and SHAP across 304 OpenML datasets. Consistent with their results, we observed that LIME had faster mean computation times than KernelSHAP (LR, SVM, ANN, and VE) but was slightly slower than TreeSHAP (RF, GB). A second important result that was similar to that of Doumard et al. (2023). Specifically, SHAP was consistently more robust than LIME; this indicates that SHAP is less variable with respect to small changes in the input features. SHAP also had higher readability scores and generally higher clusterability scores than LIME, indicating that SHAP has visually clearer dependence plots and incorporates interactions between features better than LIME. Our results differed from those in the previous study in that they found that LIME generally had the lowest AUFIC, whereas we observed that LIME had higher average AUFIC values indicating that it assigns higher values to fewer features. While our findings generally align with those of Doumard et al., our study highlights the advantages of using SHAP over LIME, specifically within the context of a clinical neurodegenerative dataset, rather than across a diverse array of data sources. Furthermore, as detailed below, we evaluate the similarities between the two XAI algorithms, and when combined with these results, this assessment provides further guidance for which approach to choose for similar aging and neurodegeneration studies.

Next, we compared the two XAI algorithms in terms of their similarity. We observed that LIME and SHAP ranked features similarly in terms of composition ratio and identified similar numbers of leading predictors. However, between the two approaches typically only around half of the features had matching directions of influence. This means that while the algorithms agree on which features are influential, they sometimes disagree on how the feature impacts the prediction. We also noticed that the two XAI algorithms produced similar results most often between LR and SVM. This can be explained by both being linear approaches and thus there is little difference in using an algorithm that can utilize feature interactions such as SHAP over a linear approach like LIME. Considering both the performance and similarity results of the two XAI algorithms, we recommend using SHAP as it outperforms LIME across multiple metrics while identifying similar features as important, although with differing directions of influence. It should also be noted that while we systematically compared two frequently used XAI algorithms (LIME and SHAP) in this literature, other algorithms such as rule-based or example-based approaches (Van der Waa et al., 2021) may be able to provide additional insights.

Finally, we compared the leading predictors across pairs of supervised ML models and XAI algorithms for each cohort comparison task. In the AD vs. CU task, we observed that olfaction, sex, memory QOL, handling money, grip strength, and timed walk were leading predictors in at least 10 out of 12 pairs of ML and follow-up XAI approaches. These factors have been previously identified as symptomatically associated with AD and AD risk (Buchman et al., 2007; Jahn, 2013; Martin et al., 2008; Pike, 2016; Ries et al., 2009; Zou et al., 2016). In the MCI vs. CU task, we observed that memory QOL, sex, grip strength, pulse pressure, and self-rated health were the consistent leading predictors in at least 10 ML-XAI pairs. Previous studies have found associations between each of these factors and cognitive decline and impairment (Cui et al., 2021; Fritz et al., 2017; Meyer et al., 2017; Petersen et al., 2010b; Waldstein et al., 2007). We highlight sex, as it has been identified as a crucial factor in AD risk and diagnosis (Subramaniapillai et al., 2021; Tierney et al., 2017). In the present case, however, the identification of sex as a leading predictor in both the AD vs. CU and MCI vs. CU tasks may be related to an imbalance in the distribution of sex within and across the present cohorts [AD (30% female), MCI (49% female), SCI (83% female), and CU (82% female)]. In the SCI vs. CU task, we observed memory QOL and lymphocytes number as leading predictors in at least 10 of the ML-XAI combinations. Some prior work has also observed associations between these factors and SCI (Hill et al., 2017; Kalelioglu et al., 2017).

Although we noted several limitations of this research in the preceding section, we identify four additional limitations attributable to the specific dataset we used. We evaluated and compared different ML algorithms using a dataset with a wide range of 102 features spanning 17 morbidity domains. First, these features were indeed derived from multiple modalities (a strength) but their large number and alignment with morbidity and deficit domains restricted our ability to combine or compare across datasets. We were thus focused on a comprehensive approach to a single dataset. Second, the current dataset included multiple cohorts in the AD spectrum (a strength), but the sample sizes within these cohorts were different and relatively small. Third, as noted above the sex distribution within cohorts was unbalanced, but we did find useful results. Fourth, although we thoroughly compared the ML algorithms using this dataset, we were unable to fully capture sample diversity, potentially limiting the generalizability of our results.

## 5 Conclusion

The present study evaluated three RGs using a discrete clinical neurodegeneration dataset: (1) compare the performance of seven common and promising ML algorithms in discriminating between four clinical cohorts which represent progression along an the AD spectrum (CU, SCI, MCI, AD); (2) assess the relative performance and similarity between two commonly used XAI model interpretation techniques; and (3) informally evaluate the leading predictors from each combination of ML and XAI approach and discuss the predictors that are most commonly identified as having a notable influence on model predictions.

In comparing ML models, we observed that the decision-tree-based methods, RF and GB, outperformed the other models in all discrimination tasks except in the AD vs. CU setting where SL performed the best in all metrics except recall. Notably, SL is an ensemble method, and it is likely that either RF or GB were used in the final SL model. ML performance was best in the AD vs. CU task and degraded when the compared cohorts were closer to each other along the AD spectrum. The worst overall performance was seen in the multi-class setting, which is expected since the number of classes is double that of the binary tasks.

From our systematic comparative analyses, we observed RF, GB, and SL consistently outperforming other commonly used ML approaches within neurodegeneration research and therefore we recommend these models for use with the COMPASS-ND dataset or similar clinical neurodegenerative datasets. However, it should be noted that the extent to which these results may generalize to other types of data (e.g., neuroimaging) or other clinical datasets is unknown. The present results do, however, suggest that RF or GB work well as an initial model for discrete clinical neurodegenerative research, with GB slightly outperforming RF and may be a better choice if a researcher is limited to a single model with similar data. Further work is required to support these algorithms in other contexts such as different datasets, cohort comparisons, or regression tasks. The present approach can be adapted to multiple variations of these dimensions.

In the subsequent comparison of XAI algorithms, we observed that TreeSHAP, a model-specific version of SHAP that works with RF and GB, outperformed LIME according to almost all metrics. In assessing the similarity between the algorithms, we observed that both LIME and SHAP typically agreed on which features have high influence on predictions but differed in the direction of influence. We also noted that LIME and SHAP performed most similarly when applied to linear approaches (LR and SVM). From our observations, we recommend combining TreeSHAP with RF or GB to highlight useful and potentially clinically relevant features. We also recommend viewing the results of XAI algorithms with a critical eye and caution against using them as definitive evidence for causal relationships.

Comparing the leading predictors across combinations of ML and XAI approaches, we observed that sex and grip strength were identified by all combinations in both the AD vs. CU and MCI vs. CU tasks. Olfaction and memory QOL were also identified in the AD vs. CU task, and pulse pressure was identified in the MCI vs. CU task. Memory QOL and lymphocytes number were shared across most combinations within the SCI vs. CU task. The variance in leading predictors observed across various combinations of ML models and XAI algorithms calls attention to the risk of presuming a strong correlation between identified features and a cohort. These results emphasize that XAI algorithms offer one among several possible explanations, highlighting the need for critical consideration before making further conclusions.

## Data Availability

The data analyzed in this study is subject to the following licenses/restrictions: The authors will make data available upon reasonable request to the corresponding author and approval by the Canadian Consortium on Neurodegeneration in Aging Publications and Data Access Committee. Requests to access these datasets should be directed to ccna@ladydavis.ca.
