# Supplementary material for: Comparing machine learning classifier models in discriminating cognitively unimpaired older adults from three clinical cohorts in the Alzheimer’s disease spectrum: demonstration analyses in the COMPASS-ND study
Source: Front Aging Neurosci. 2025 Mar 4;17:1542514. doi: 10.3389/fnagi.2025.1542514 (PMC11913811; doi:10.3389/fnagi.2025.1542514)
Supplement: Supplementary file 1 [file Table_1.docx]

Supplementary Material

# Supplementary Figures and Tables

## Supplementary Figures

**
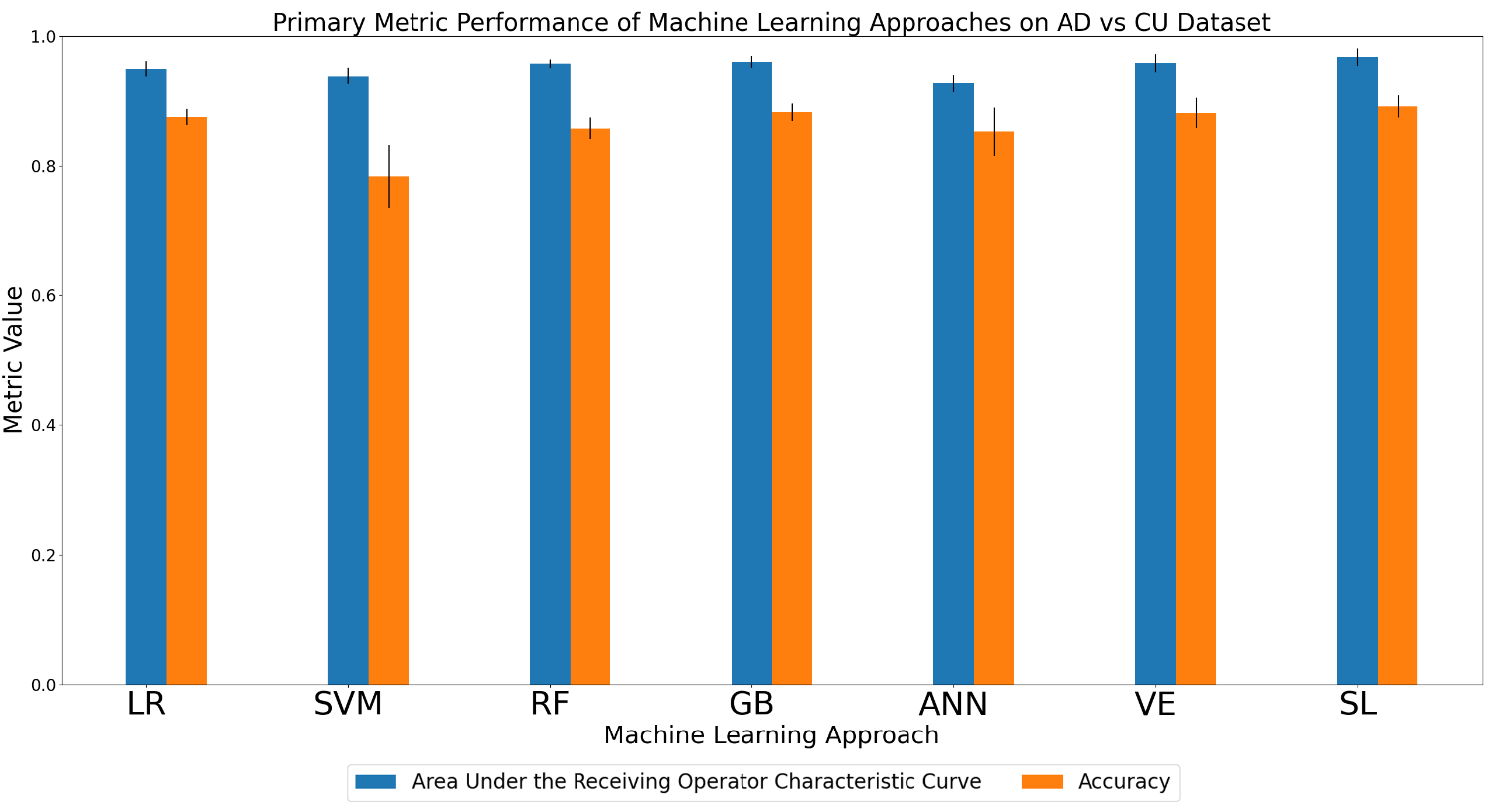
**

**Supplementary Figure 1.** Comparison of mean AUC and accuracy of ML approaches on the AD vs CU dataset over 10 trials of 5-fold cross-validation. Error bars represent the standard deviation. Cohort and model acronyms identified in the text.

**
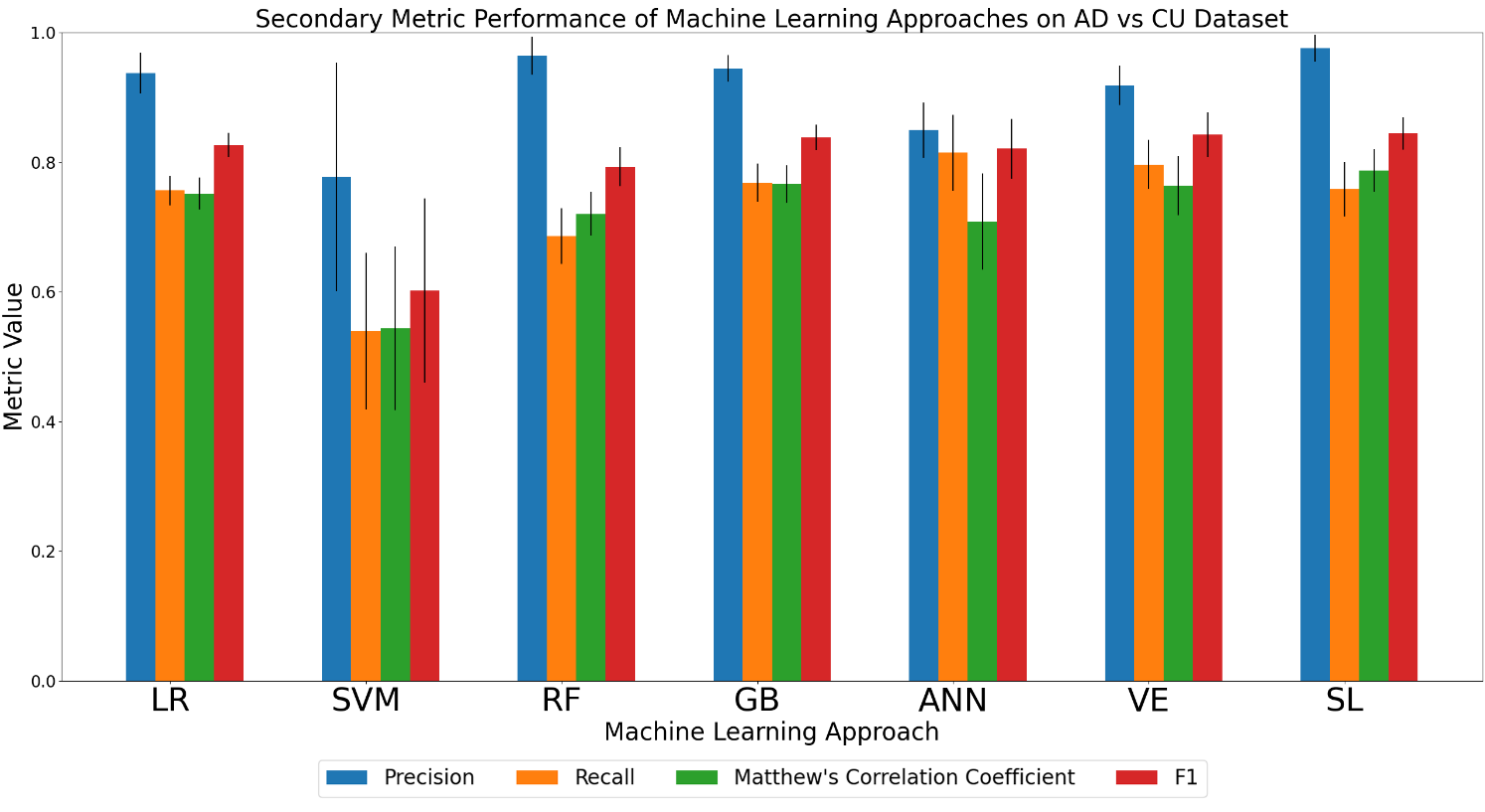
**

**Supplementary Figure 2.** Comparison of mean precision, recall, MCC and F1 of ML approaches on the AD vs CU dataset over 10 trails of 5-fold cross-validation. Error bars represent the standard deviation. Cohort and model acronyms identified in the text.

**
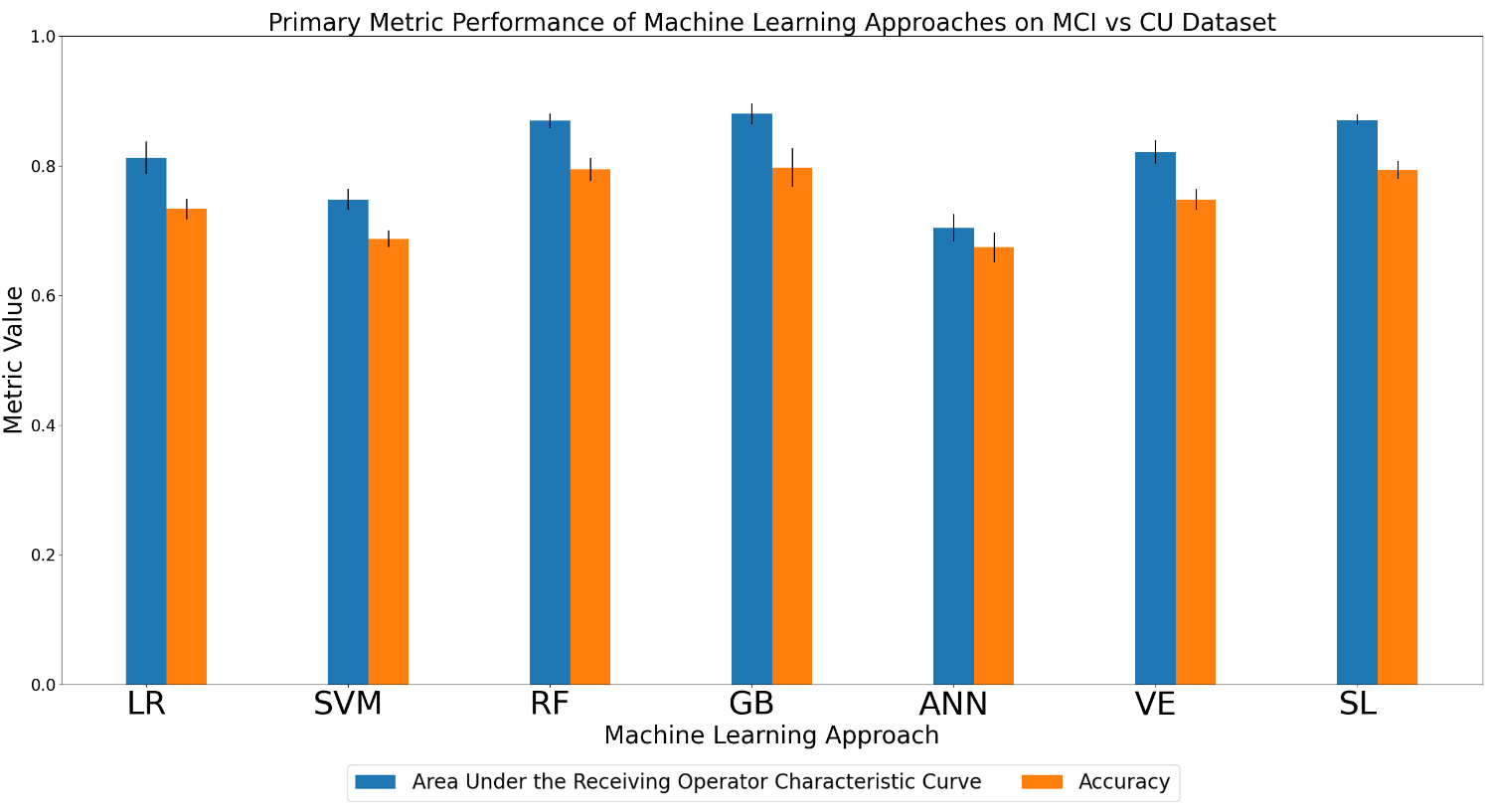
**

**Supplementary Figure 3.** Comparison of mean AUC and accuracy of ML approaches on the MCI vs CU dataset over 10 trials of 5-fold cross-validation. Error bars represent the standard deviation. Cohort and model acronyms identified in the text.

**
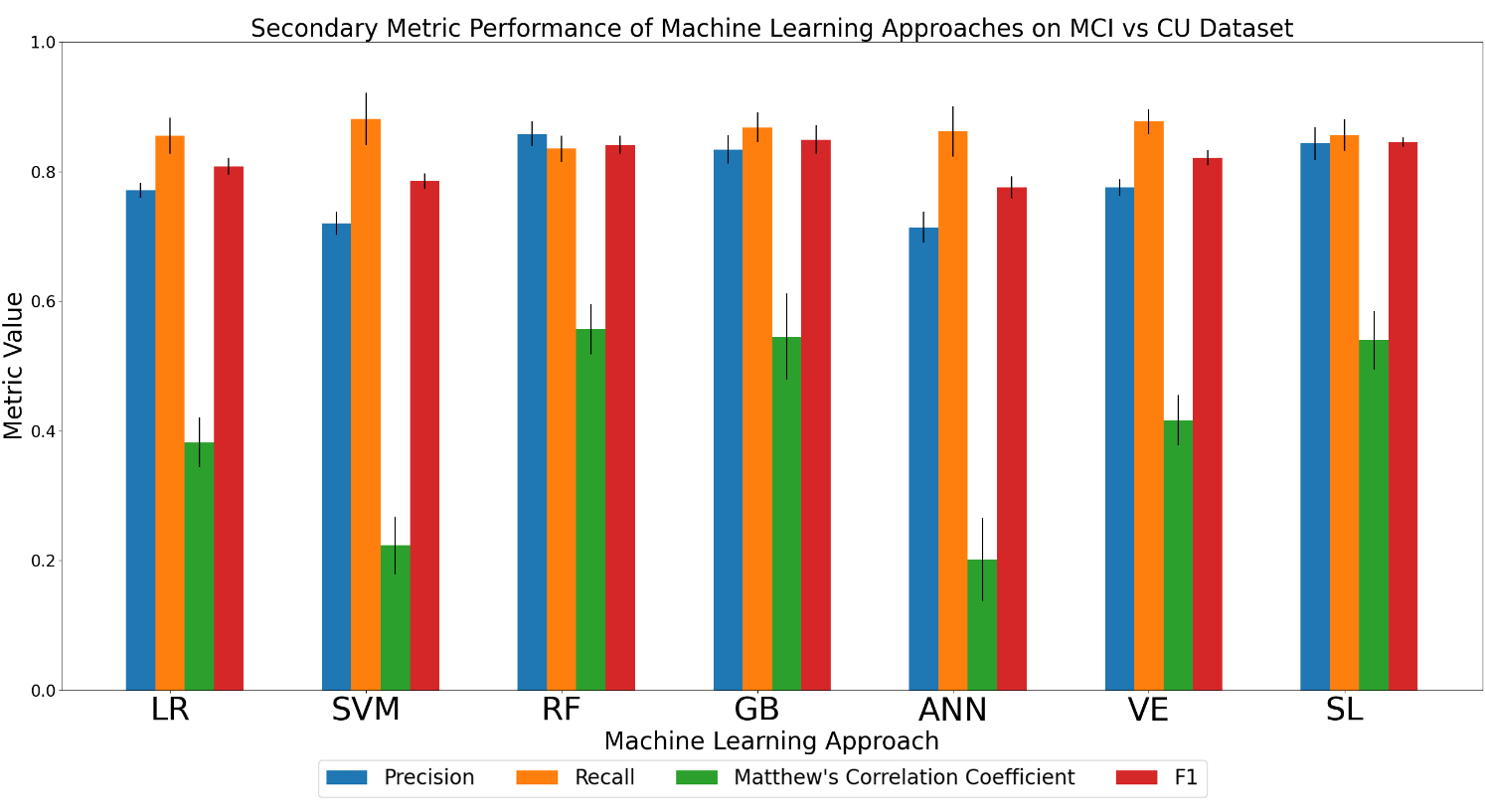
**

**Supplementary Figure 4.** Comparison of mean precision, recall, MCC and F1 of ML approaches on the MCI vs CU dataset over 10 trials of 5-fold cross-validation. Error bars represent the standard deviation. Cohort and model acronyms identified in the text.

**
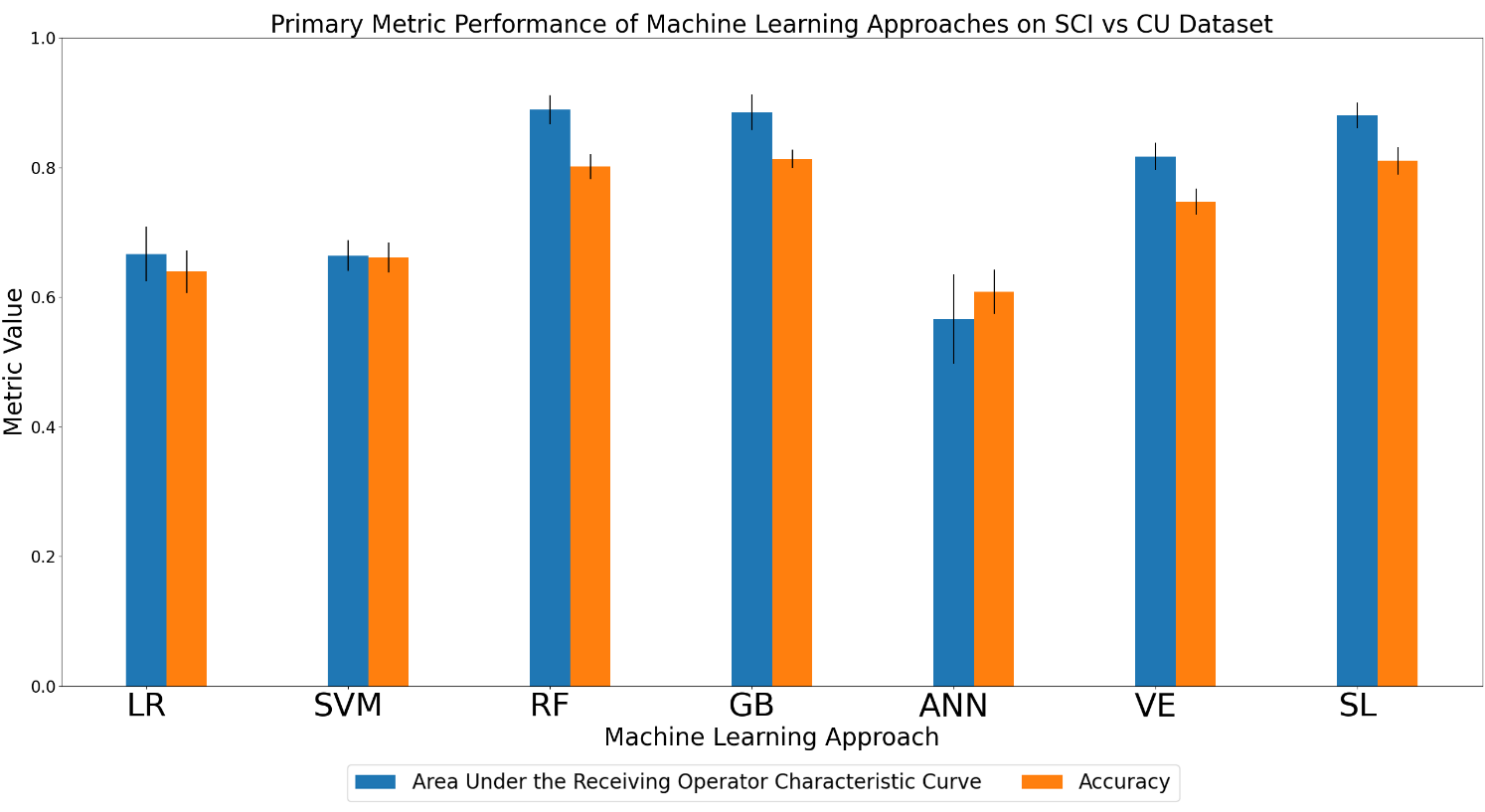
**

**Supplementary Figure 5.** Comparison of mean AUC and accuracy of ML approaches on the SCI vs CU dataset over 10 trials of 5-fold cross-validation. Error bars represent the standard deviation. Cohort and model acronyms identified in the text.

**
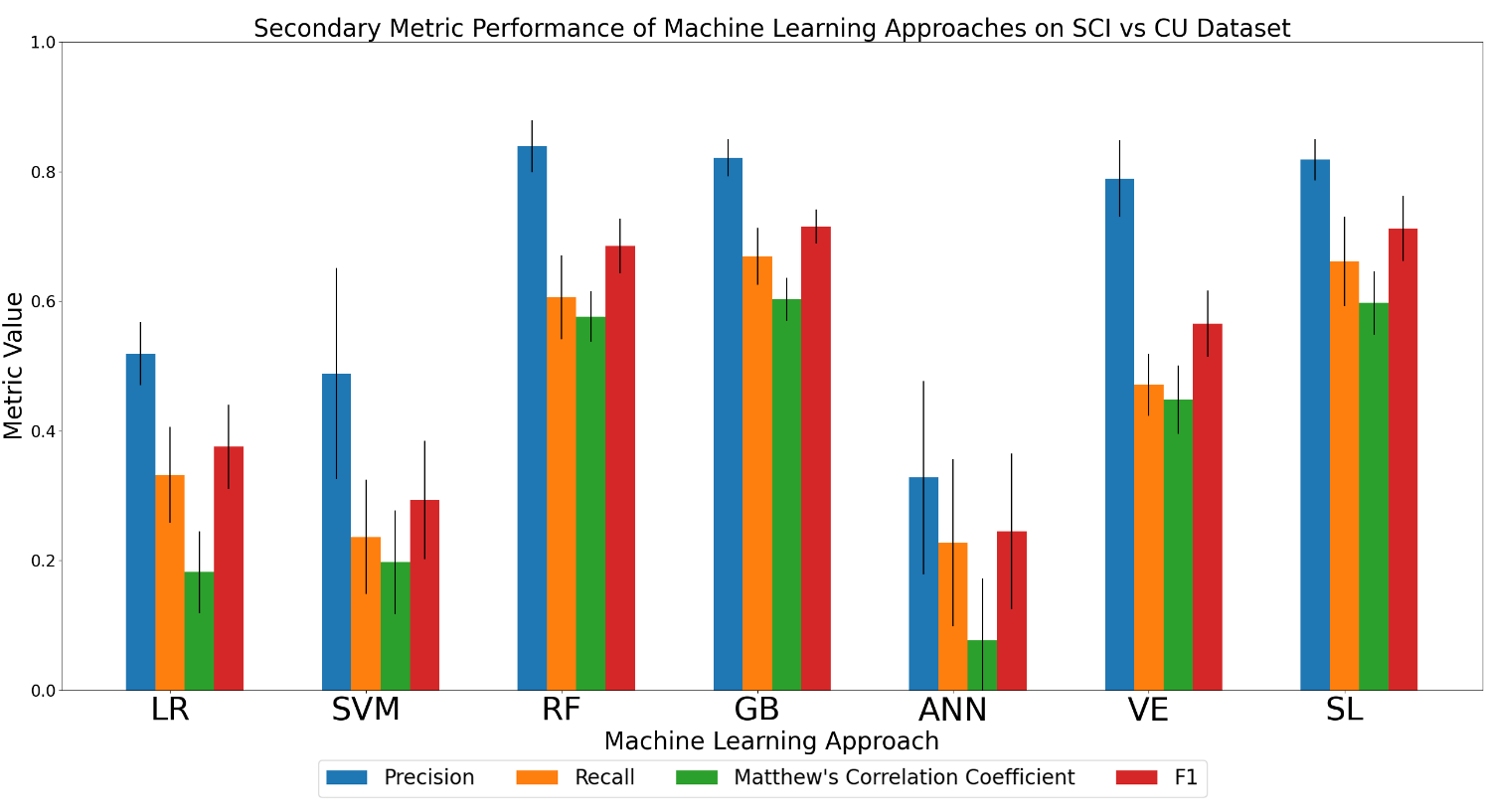
**

**Supplementary Figure 6.** Comparison of mean precision, recall, MCC and F1 of ML approaches on the SCI vs CU dataset over 10 trials of 5-fold cross-validation. Error bars represent the standard deviation. Cohort and model acronyms identified in the text.


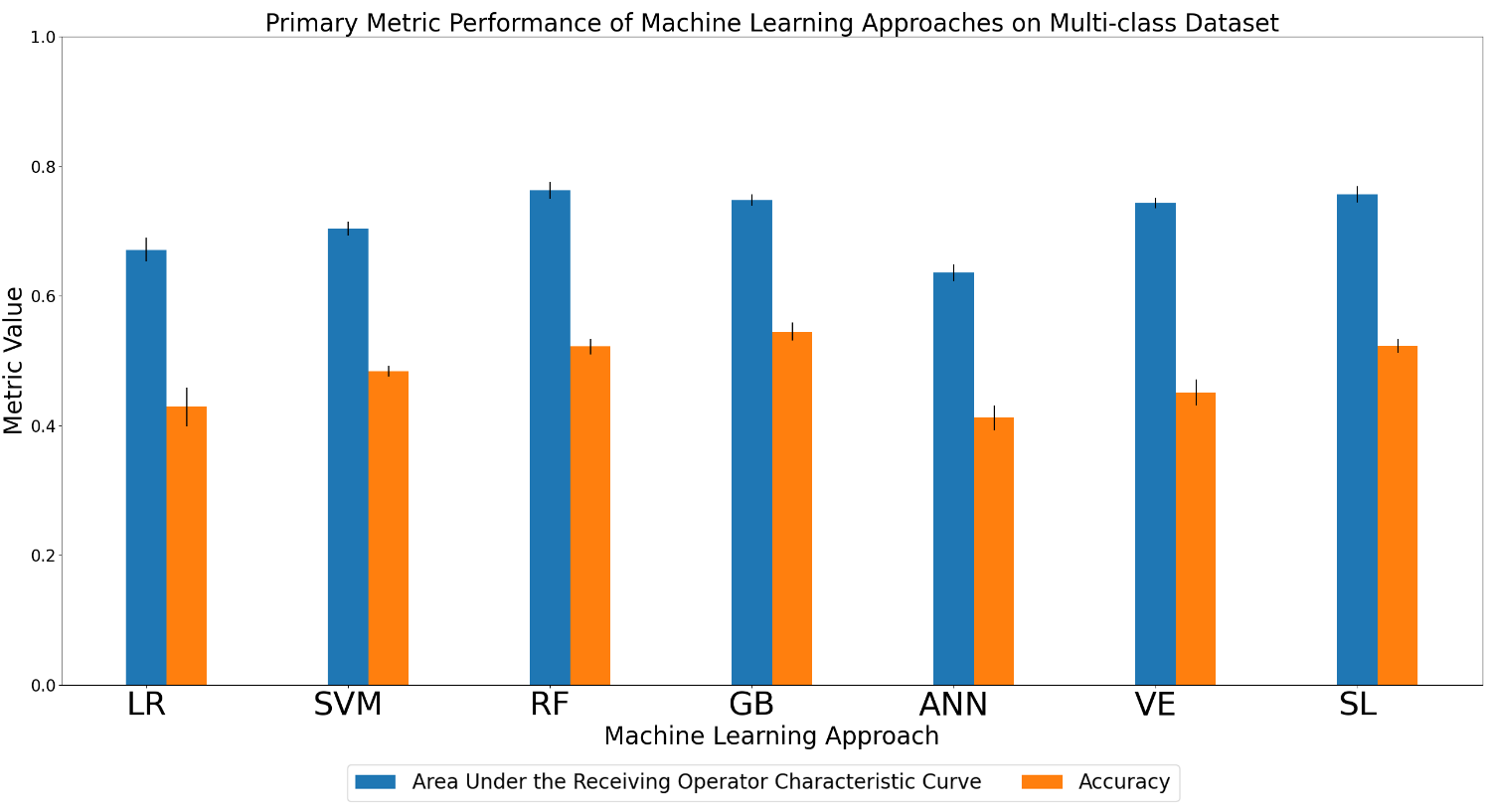


**Supplementary Figure 7.** Comparison of mean AUC and accuracy of ML approaches on the AD vs MCI vs SCI vs CU dataset over 10 trials of 5-fold cross-validation. Error bars represent the standard deviation. Cohort and model acronyms identified in the text.

**
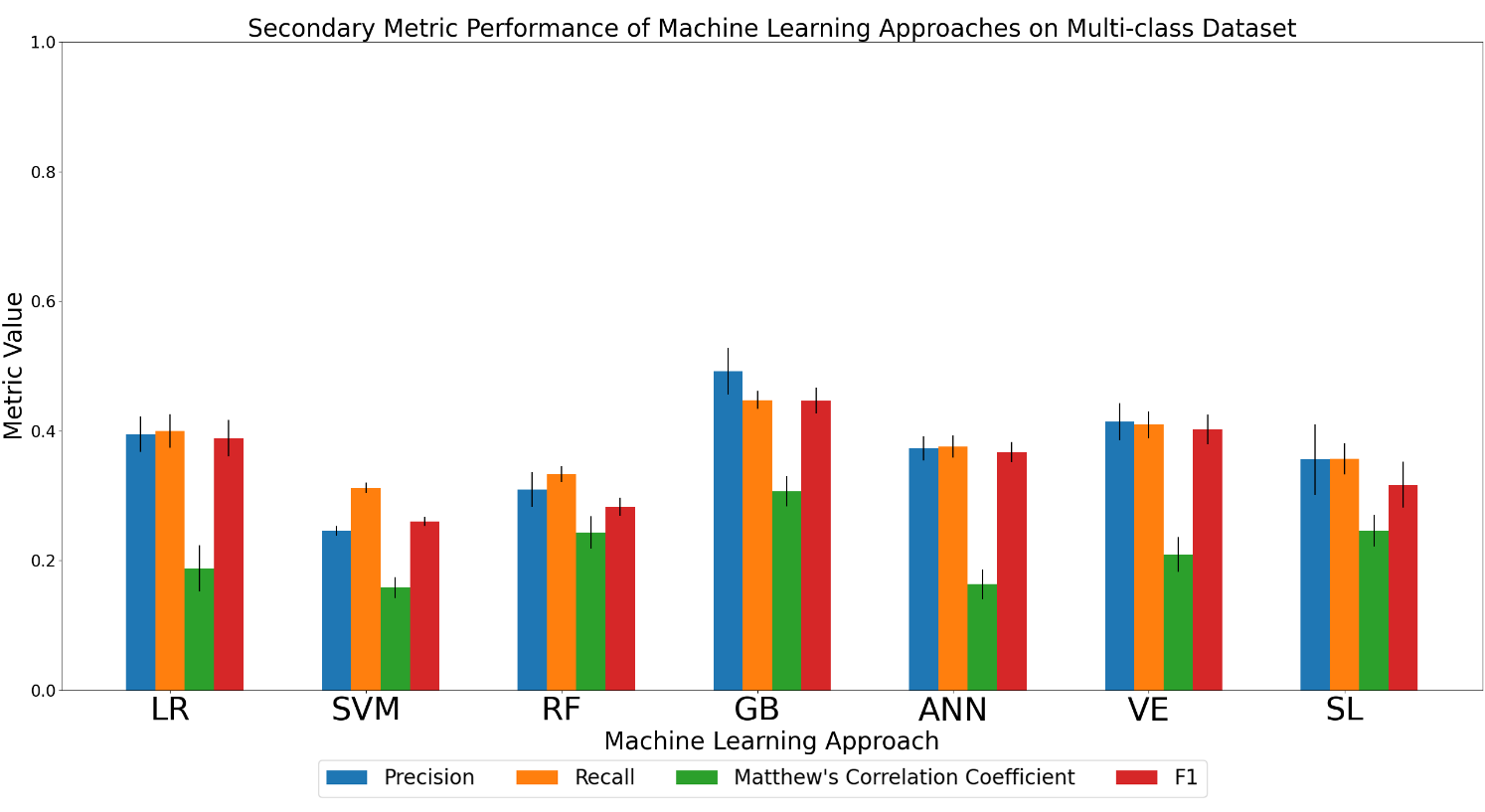
Supplementary Figure 8.** Comparison of mean precision, recall, MCC and F1 of ML approaches on the AD vs MCI vs SCI vs CU dataset over 10 trials of 5-fold cross-validation. Error bars represent the standard deviation. Cohort and model acronyms identified in the text.

## Supplementary Tables

**Supplementary Table 1.** Response scales for frailty-related indicators eligible for inclusion in machine learning analysis (*n* = 83)

| **Domain** | **Indicator** | | | **Response Scale** |  |
| --- | --- | --- | --- | --- | --- |
| **IADL** | Getting to places beyond walking distance | | 0 = without help; 0.5 = with some help; 1 = completely unable | |  |
|  | Going shopping for groceries or clothes | |  |  |  |
|  | Preparing meals | |  |  |  |
|  | Doing housework | |  |  |  |
|  | Taking medication | |  | |  |
|  | Handling money | |  | |  |
| **ADL** | Trouble getting to bathroom in time | | 0 = no; 1 = yes | |  |
| **Physical activity** | Physical activity (PASE score) | | ﻿0 = ≥ 64 males; ≥ 52 females  1 = < 64 males; < 52 females | |  |
| **Mobility** | Self-reported balance | | 0 = very good; 0.5 = pretty good; 1 = very poor | |  |
|  | Balance confidence (ABC score) | | 0 = high; 0.5 = moderate; 1 = low | |  |
|  | Timed walk (averaged over three 6m trials) | | 0 = > 1m/s;1 = < 1m/s | |  |
|  | Falls in the past year | | 0 = no; 1 = yes | |  |
| **QoL** | Physical health | | 0 = excellent; 0.33 good; 0.66 = fair; 1 = poor | |  |
|  | Energy | |  |  |  |
|  | Mood | |  | |  |
|  | Memory | |  | |  |
|  | Ability to do chores around the house | |  | |  |
|  | Ability to do things for fun | |  | |  |
| **Anthropometric measures** | Waist-to-hip ratio | | 0 = < 0.85 females; < 0.96 males 1 = > 0.85 females, > 0.96 males | |  |
|  | Waist circumference (cm) | | 0 < 88 females; < 102 males  1 > 88 females; > 102 males | |  |
|  | Body mass index (kg/m^2^) | | 0 = 18.5-25; 0.5 = 25 to < 30;  1 = < 18.5 or ≥ 30 | |  |
|  | Unintentional weight loss | | 0 = no; 1 = yes | |  |
| **Sensory function** | Self-reported eyesight | | 0 = excellent; 0.25 = very good; 0.50 = good; 0.75 = fair; 1 = poor or non-existent | |  |
|  | Self-reported hearing | |  |  |  |
|  | Hearing handicap (HHIE score) | | 0 = no hearing handicap; 0.5 = mild/moderate handicap; 1 = significant handicap | |  |
|  | Olfaction (B-SIT score) | | 0 = > 11; 1 = < 10 | |  |
|  | Visual contrast sensitivity (Mars Letter Contrast Sensitivity Test) | | 0 = normal; 0.5 = moderate; 1 = severe impairment | |  |
|  | Cataracts | | 0 = no; 1 = yes | |  |
|  | Macular degeneration | | 0 = no; 1 = yes | |  |
| **Sleep** | Sleep duration (PSQI score) | | 0 = none; 0.33 = slight problem; 0.66 = somewhat a problem; 1 = big problem | |  |
|  | Sleep efficiency (PSQI score) | |  |  |  |
|  | Sleep disturbances (PSQI score) | |  |  |  |
|  | Daytime dysfunction (PSQI score) | |  |  |  |
|  | Sleep latency (PSQI score) | |  |  |  |
|  | Sleep medication (PSQI score) | |  |  |  |
|  | Self-reported sleep quality | | 0 = very good; 0.33 = fairly good; 0.66 = fairly bad; 1 = very bad | |  |
| **Functional indicators** | Grip strength (averaged over three trials) | | Males: for BMI ≤ 24, GS ≤ 29; for BMI 24.1-28, GS ≤ 30; for BMI > 28, GS ≤ 32  Females: for BMI ≤ 23, GS ≤ 17; for BMI 23.1-26, GS ≤ 17.3; for BMI 26.1-29, GS ≤ 18; for BMI > 29, GS ≤ 21 | |  |
|  | Pulse pressure (mmHg) | | 0 = 32.13-63.90; 0.5 = 64-75.9; 1 = < 32.12 or 76+ | |  |
|  | Resting heart rate (bpm) | | 0 = 60-99; 1 = < 60 or 100 < | |  |
| **Exhaustion** | Everything an effort  Could not get going | | 0 = rarely or none of the time; 0.25 = some or a little of the time; 0.75 = a moderate amount; 1 = most of the time | |  |
| **Self-reported health** | | Current health | | 0 = very good; 0.25 = good; 0.50 = average; 0.75 = poor; 1 = very poor | |
| **Cardiorespiratory health** | Chronic respiratory condition | | 0 = no; 1 = yes | |  |
|  | Shortness of breath | |  | |  |
|  | Sleep breathing disorder | |  | |  |
|  | High blood pressure or hypertension | |  | |  |
|  | Atrial fibrillation or irregular heartbeat | |  | |  |
|  | Heart attack, congestive heart failure | |  | |  |
|  | Peripheral vascular disease | |  | |  |
|  | Mini-stroke or TIA | |  | |  |
|  | Hyperlipidemia | |  | |  |
| **Clinical symptoms or diseases** | Episodes of fainting | | 0 = no; 1 = yes | |  |
|  | Orthostatic blood pressure drop | |  | |  |
|  | Vertigo or dizziness | |  | |  |
|  | Type II diabetes | | 0 = no; 0.5 = borderline/high blood sugar; 1 = type I or II diabetes | |  |
|  | Polypharmacy | | 0 = 0-4 medications; 0.5 = 5-7 medications; 1 = 8+ medications; 2 = 14+ medications | |  |
|  | Osteoarthritis | | 0 = no; 1 = yes | |  |
|  | Hypothyroidism | | 0 = no; 1 = yes | |  |
|  | Osteoporosis | | 0 = no; 1 = yes | |  |
|  | Stomach ulcers | | 0 = no; 1 = yes | |  |
|  | Irritable bowel syndrome | | 0 = no; 1 = yes | |  |
|  | Chronic constipation | | 0 = no; 1 = yes | |  |
|  | Urinary incontinence | | 0 = no; 1 = yes | |  |
|  | Cancer | | 0 = no; 1 = yes | |  |
| **Emotional wellbeing** | Major depressive disorder | | 0 = no; 1 = yes | |  |
|  | Generalized anxiety disorder | |  | |  |
| **Oral health and nutritional factors** | Self-reported appetite | | 0 = very good; 0.33 = good; 0.66 = fair; 1 = poor | |  |
|  | Coughing, choking, pain when swallowing | | 0 = never; 0.33 = rarely; 0.66 = sometimes; 1 = often or always | |  |
|  | Self-reported mouth health | | 0 = excellent; 0.25 = very good; 0.50 = good; 0.75 = fair; 1 = poor or non-existent | |  |
|  | Eating discomfort due to mouth problems | | 0 = never; 0.33 = rarely; 0.66 = sometimes; 1 = often or always | |  |
|  | Avoid eating particular food due to mouth | |  | |  |
| **Fluid biomarkers** | Hemoglobin | | 0 = inside established reference range; 1 = outside established reference range | |  |
|  | HbA1c | |  |  |  |
|  | Mean corpuscular hemoglobin concentration | |  |  |  |
|  | Mean corpuscular hemoglobin | |  |  |  |
|  | Mean corpuscular volume | |  | |  |
|  | White blood cell count | |  | |  |
|  | Red blood cell count | |  | |  |
|  | Red cell distribution width | |  | |  |
|  | Number of lymphocytes | |  | |  |
|  | Number of neutrophils | |  | |  |
|  | Hematocrit | |  | |  |
| **Sex** | Male or Female | | 0 = male; 1 = female | |  |

# *Note.* Abbreviations: IADL, instrumental activities of daily living; ADL, basic activities of daily living; QoL, quality of life; PASE, adapted Physical Activity Scale for the Elderly (Washburn et al., 1993); ABC, Activities-specific Balance Confidence Scale (Powell et al., 1995); HHIE, Hearing Handicap Inventory for the Elderly— screening version (Ventry & Weinstein, 1982); B-SIT, Brief Smell Identification Test (Menon et al., 2013); PSQI, Pittsburgh Sleep Quality Index (Buysse et al., 1989); TIA, transient ischemic attack; HbA1c, glycated hemoglobin.d

**Supplementary Table 2.** Supervised ML algorithm hyperparameters.

| **Logistic Regression** | |
| --- | --- |
| solver | **‘**lbfgs’, ‘liblinear’ |
| penalty | ‘none’, ‘l1’, ‘l2’ |
| C | 0.1, 1, 10 |
| **Support Vector Machine** | |
| kernel | ‘linear’, ‘rbf’, ‘poly’ |
| C | 0.1, 1, 10, 100 |
| gamma | 0.1, 1, 10 |
| degree | 2, 3, 4, 5, 6 |
| **Random Forest** | |
| n_estimators | 100, 250, 500, 1000 |
| criterion | ‘gini’, ‘log_loss’ |
| max_depth | 3, 6, 9, 18, None |
| max_features | ‘sqrt’, ‘log2’, None |
| **Gradient-boosted Trees** | |
| n_estimators | 100, 250, 500, 1000 |
| criterion | ‘friedman_mse’, ‘squared_error' |
| max_depth | 3, 6, 9, 18, None |
| max_features | ‘sqrt’, ‘log2’, None |
| **Artificial Neural Network** | |
| hidden_layer_sizes | (10,), (20,), (50,), (10,10), (20,20), (50,50) |
| activation | ‘identity’, ‘logistic’, ‘tanh’, ‘relu’ |
| solver | ‘lbfgs’, ‘sgd’, ‘adam’ |
| learning_rate | ‘constant’, ‘invscaling’, ‘adaptive’ |

**Supplementary Table 3.** Supervised ML algorithm hyperparameters used for evaluating XAI approaches on the **AD vs CU** dataset. Selected hyperparameters are those with the highest average AUC over 5-fold cross-validation on the dataset.

| **Logistic Regression** | |
| --- | --- |
| solver | **‘**lbfgs’ |
| penalty | ‘l2’ |
| C | 0.1 |
| **Support Vector Machine** | |
| kernel | ‘rbf’ |
| C | 1 |
| gamma | 0.1 |
| degree | 2 |
| **Random Forest** | |
| n_estimators | 100 |
| criterion | ‘log_loss’ |
| max_depth | 6 |
| max_features | ‘sqrt’ |
| **Gradient-boosted Trees** | |
| n_estimators | 100 |
| criterion | ‘friedman_mse’ |
| max_depth | 18 |
| max_features | ‘log2’ |
| **Artificial Neural Network** | |
| hidden_layer_sizes | (20,) |
| activation | ‘relu’ |
| solver | ‘lbfgs’ |
| learning_rate | ‘invscaling’ |

*Note.* Abbreviations: ML, Machine Learning; XAI, eXplainable Artificial Intellignece; AD, Alzheimer’s Disease; CU, Cognitively Unimpaired.

**Supplementary Table 4.** Supervised ML algorithm hyperparameters selected for evaluating XAI approaches on the **MCI vs CU** dataset. Selected hyperparameters are those with the highest average AUC over 5-fold cross-validation on the dataset.

| **Logistic Regression** | |
| --- | --- |
| solver | ‘liblinear’ |
| penalty | ‘l1’ |
| C | 1 |
| **Support Vector Machine** | |
| kernel | ‘linear’ |
| C | 0.1 |
| gamma | 0.1 |
| degree | 2 |
| **Random Forest** | |
| n_estimators | 250 |
| criterion | ‘log_loss’ |
| max_depth | 9 |
| max_features | ‘sqrt’ |
| **Gradient-boosted Trees** | |
| n_estimators | 100 |
| criterion | ‘friedman_mse’ |
| max_depth | 3 |
| max_features | ‘sqrt’ |
| **Artificial Neural Network** | |
| hidden_layer_sizes | (10,) |
| activation | ‘identity’ |
| solver | ‘adam’ |
| learning_rate | ‘adaptive’ |

*Note.* Abbreviations: ML, Machine Learning; XAI, eXplainable Artificial Intellignece; MCI, Mild Cognitive Impairment; CU, Cognitively Unimpaired.

**Supplementary Table 5.** Supervised ML algorithm hyperparameters selected for evaluating XAI approaches on the **SCI vs CU** dataset. Selected hyperparameters are those with the highest average AUC over 5-fold cross-validation on the dataset.

| **Logistic Regression** | |
| --- | --- |
| solver | ‘lbfgs’ |
| penalty | ‘l2’ |
| C | 0.1 |
| **Support Vector Machine** | |
| kernel | ‘poly’ |
| C | 1 |
| gamma | 0.2 |
| degree | 2 |
| **Random Forest** | |
| n_estimators | 100 |
| criterion | ‘gini’ |
| max_depth | 18 |
| max_features | ‘sqrt’ |
| **Gradient-boosted Trees** | |
| n_estimators | 1000 |
| criterion | ‘squared_error’ |
| max_depth | 6 |
| max_features | ‘sqrt’ |
| **Artificial Neural Network** | |
| hidden_layer_sizes | (50, 50) |
| activation | ‘tanh’ |
| solver | ‘sgd’ |
| learning_rate | ‘constant’ |

*Note.* Abbreviations: ML, Machine Learning; XAI, eXplainable Artificial Intellignece; SCI, Subjective Cognitive Impairment; CU, Cognitively Unimpaired.

**Supplementary Table 6.** Best performing supervised ML algorithm hyperparameters for the **AD vs MCI vs** **SCI vs CU** dataset. Best hyperparameters are those with the highest average AUC over 5-fold cross-validation on the dataset.

| **Logistic Regression** | |
| --- | --- |
| solver | ‘lbfgs’ |
| penalty | ‘none’ |
| C | 0.1 |
| **Support Vector Machine** | |
| kernel | ‘linear’ |
| C | 0.1 |
| gamma | 0.1 |
| degree | 2 |
| **Random Forest** | |
| n_estimators | 100 |
| criterion | ‘gini’ |
| max_depth | 3 |
| max_features | ‘sqrt’ |
| **Gradient-boosted Trees** | |
| n_estimators | 100 |
| criterion | ‘friedman_mse’ |
| max_depth | 3 |
| max_features | ‘sqrt’ |
| **Artificial Neural Network** | |
| hidden_layer_sizes | (10,) |
| activation | ‘identity’ |
| solver | ‘lbfgs’ |
| learning_rate | ‘constant’ |

*Note.* Abbreviations: ML, Machine Learning; XAI, eXplainable Artificial Intellignece; SCI, Subjective Cognitive Impairment; CU, Cognitively Unimpaired.

**Supplementary Table 7.** Mean model performance of **AD vs CU** discrimination task over 10 trials of 5-fold cross-validation.

| **Model** | **AUC** | **Accuracy** | **Precision** | **Recall** | **MCC** | **F1** |
| --- | --- | --- | --- | --- | --- | --- |
| **LR** | 0.95 (0.01) | 0.87 (0.01) | 0.94 (0.03) | 0.76 (0.02) | 0.75 (0.03) | 0.83 (0.02) |
| **SVM** | 0.94 (0.01) | 0.78 (0.05) | 0.78 (0.18) | 0.54 (0.12) | 0.54 (0.13) | 0.60 (0.14) |
| **RF** | 0.96 (0.01) | 0.86 (0.02) | 0.96 (0.03) | 0.69 (0.04) | 0.72 (0.03) | 0.79 (0.03) |
| **GB** | 0.96 (0.01) | 0.88 (0.01) | 0.95 (0.02) | 0.77 (0.03) | 0.77 (0.03) | **0.84 (0.02)** |
| **ANN** | 0.93 (0.01) | 0.85 (0.04) | 0.85 (0.04) | **0.81 (0.06)** | 0.71 (0.08) | 0.82 (0.05) |
| **VE** | 0.96 (0.01) | 0.88 (0.02) | 0.92 (0.03) | 0.80 (0.04) | 0.76 (0.05) | **0.84 (0.04)** |
| **SL** | **0.97 (0.01)** | **0.89 (0.02)** | **0.98 (0.02)** | 0.76 (0.04) | **0.79 (0.03)** | **0.84 (0.03)** |

*Note.* Results are presented as mean (standard deviation). Values in bold print identify the numerically highest performance by a model for each metric, with the qualification that other model performances are often clustered at similar levels. Abbreviations: AD, Alzheimer’s Disease; CU, Cognitively Unimpaired; AUC, Area Under the receiving operator characteristic Curve; MCC, Matthew’s Correlation Coefficient; LR, Logistic Regression; SVM, Support Vector Machine; RF, Random Forest; GB, Gradient-Boosted trees; ANN, Artificial Neural Network; VE, Voting Ensemble; SL, Super Learner.

**Supplementary Table 8.** Mean model performance of **MCI vs CU** over 10 trials of 5-fold cross-validation.

| **Model** | **AUC** | **Accuracy** | **Precision** | **Recall** | **MCC** | **F1** |
| --- | --- | --- | --- | --- | --- | --- |
| **LR** | 0.81 (0.03) | 0.73 (0.02) | 0.77 (0.01) | 0.86 (0.03) | 0.38 (0.04) | 0.81 (0.01) |
| **SVM** | 0.75 (0.02) | 0.69 (0.01) | 0.72 (0.02) | **0.88 (0.04)** | 0.22 (0.04) | 0.79 (0.01) |
| **RF** | 0.87 (0.01) | 0.79 (0.02) | **0.86 (0.02)** | 0.84 (0.02) | **0.56 (0.04)** | 0.84 (0.01) |
| **GB** | **0.88 (0.02)** | **0.80 (0.03)** | 0.83 (0.02) | 0.87 (0.02) | 0.54 (0.07) | **0.85 (0.02)** |
| **ANN** | 0.70 (0.02) | 0.67 (0.02) | 0.71 (0.02) | 0.86 (0.04) | 0.20 (0.06) | 0.77 (0.02) |
| **VE** | 0.82 (0.02) | 0.74 (0.02) | 0.78 (0.01) | **0.88 (0.02)** | 0.42 (0.04) | 0.82 (0.01) |
| **SL** | 0.87 (0.01) | 0.79 (0.01) | 0.84 (0.03) | 0.86 (0.02) | 0.54 (0.05) | **0.85 (0.01)** |

*Note.* Results are presented as mean (standard deviation). Values in bold print identify the numerically highest performance by a model for each metric, with the qualification that other model performances are often clustered at similar levels. Abbreviations: MCI, Mild Cognitive Impairment; CU, Cognitively Unimpaired; AUC, Area Under the receiving operator characteristic Curve; MCC, Matthew’s Correlation Coefficient; LR, Logistic Regression; SVM, Support Vector Machine; RF, Random Forest; GB, Gradient-Boosted trees; ANN, Artificial Neural Network; VE, Voting Ensemble; SL, Super Learner.

**Supplementary Table 9.** Mean model performance of **SCI vs CU** over 10 trials of 5-fold cross-validation.

| **Model** | **AUC** | **Accuracy** | **Precision** | **Recall** | **MCC** | **F1** |
| --- | --- | --- | --- | --- | --- | --- |
| **LR** | 0.67 (0.04) | 0.64 (0.03) | 0.52 (0.05) | 0.33 (0.07) | 0.18 (0.06) | 0.38 (0.07) |
| **SVM** | 0.66 (0.02) | 0.66 (0.02) | 0.49 (0.16) | 0.24 (0.09) | 0.20 (0.08) | 0.29 (0.09) |
| **RF** | **0.89 (0.02)** | 0.80 (0.02) | **0.84 (0.04)** | 0.61 (0.07) | 0.58 (0.04) | 0.69 (0.04) |
| **GB** | **0.89 (0.03)** | **0.81 (0.01)** | 0.82 (0.03) | **0.67 (0.04)** | **0.60 (0.03)** | **0.72 (0.03)** |
| **ANN** | 0.57 (0.07) | 0.61 (0.03) | 0.33 (0.15) | 0.23 (0.13) | 0.08 (0.10) | 0.25 (0.12) |
| **VE** | 0.82 (0.02) | 0.75 (0.02) | 0.79 (0.06) | 0.47 (0.05) | 0.45 (0.05) | 0.57 (0.05) |
| **SL** | 0.88 (0.02) | **0.81 (0.02)** | 0.82 (0.03) | 0.66 (0.07) | **0.60 (0.05)** | 0.71 (0.05) |

*Note.* Results are presented as mean (standard deviation). Values in bold print identify the numerically highest performance by a model for each metric, with the qualification that other model performances are often clustered at similar levels. Abbreviations: SCI, Subjective Cognitive Impairment; CU, Cognitively Unimpaired; AUC, Area Under the receiving operator characteristic Curve; MCC, Matthew’s Correlation Coefficient; LR, Logistic Regression; SVM, Support Vector Machine; RF, Random Forest; GB, Gradient-Boosted trees; ANN, Artificial Neural Network; VE, Voting Ensemble; SL, Super Learner.

**Supplementary Table 10.** Mean model performance of **AD vs MCI vs** **SCI vs CU** over 10 trials of 5-fold cross-validation.

| **Model** | **AUC** | **Accuracy** | **Precision** | **Recall** | **MCC** | **F1** |
| --- | --- | --- | --- | --- | --- | --- |
| **LR** | 0.67 (0.02) | 0.43 (0.03) | 0.40 (0.03) | 0.40 (0.03) | 0.19 (0.04) | 0.39 (0.03) |
| **SVM** | 0.70 (0.01) | 0.48 (0.01) | 0.25 (0.01) | 0.31 (0.01) | 0.16 (0.02) | 0.26 (0.01) |
| **RF** | **0.76 (0.01)** | 0.52 (0.01) | 0.31 (0.03) | 0.33 (0.01) | 0.24 (0.03) | 0.28 (0.01) |
| **GB** | 0.75 (0.01) | **0.55 (0.01)** | **0.49 (0.04)** | **0.45 (0.01)** | **0.31 (0.02)** | **0.45 (0.02)** |
| **ANN** | 0.64 (0.01) | 0.41 (0.02) | 0.37 (0.02) | 0.38 (0.02) | 0.16 (0.02) | 0.37 (0.02) |
| **VE** | 0.74 (0.01) | 0.45 (0.02) | 0.41 (0.03) | 0.41 (0.02) | 0.21 (0.03) | 0.40 (0.02) |
| **SL** | **0.76 (0.01)** | 0.52 (0.01) | 0.36 (0.05) | 0.36 (0.02) | 0.25 (0.02) | 0.32 (0.04) |

*Note.* Results are presented as mean (standard deviation). Values in bold print identify the numerically highest performance by a model for each metric, with the qualification that other model performances are often clustered at similar levels. Abbreviations: AD, Alzheimer’s Disease; MCI, Mild Cognitive Impairment; SCI, Subjective Cognitive Impairment; CU, Cognitively Unimpaired; AUC, Area Under the receiving operator characteristic Curve; MCC, Matthew’s Correlation Coefficient; LR, Logistic Regression; SVM, Support Vector Machine; RF, Random Forest; GB, Gradient-Boosted trees; ANN, Artificial Neural Network; VE, Voting Ensemble; SL, Super Learner.

**Supplementary Table 11.** Inter-explainer comparison of LIME and SHAP for each approach on the **AD vs CU** dataset

| **Model** | **# Matching Directions (%)**  **[0,74] ꜛ** | **Top 10 Composition Overlap**  **[0,10] ꜛ** | **Mean Absolute Composition Difference**  **[0,100] ꜜ** | **Concordance Index**  **[0,1] ꜛ** | **Number of Leading Predictors for LIME**  **[0,50]** | **Number of Leading Predictors for SHAP**  **[0,50]** |
| --- | --- | --- | --- | --- | --- | --- |
| **LR** | 37 (50%) | **9** | **0.27** | **0.91** | 9 | 8 |
| **SVM** | 34 (46%) | **9** | 0.37 | 0.86 | 10 | 5 |
| **RF** | 39 (53%) | **9** | 0.55 | 0.77 | 14 | 14 |
| **GB** | 33 (45%) | 8 | 0.52 | 0.82 | 12 | 14 |
| **ANN** | **44 (59%)** | **9** | 0.38 | 0.84 | 13 | 11 |
| **VE** | 31 (42%) | 8 | 0.35 | 0.87 | 9 | 7 |
| **Avg.** | 36.33 (49%) | 8.66 | 0.41 | 0.84 | 11.16 | 9.83 |

*Note.* The ranges of the metrics are displayed beneath the metric name for each column. ꜛ indicates that higher values on the corresponding metric denote better performance. ꜜ indicates that lower values on the corresponding metric denote better performance. Values in bold print identify the best performance by a model for each metric. Abbreviations: LIME, Local Interpretable Model agnostic Explanation; SHAP, SHapley Additive exPlanations; AD, Alzheimer’s Disease; CU, Cognitively Unimpaired; LR, Logistic Regression; SVM, Support Vector Machine; RF, Random Forest; GB, Gradient-Boosted trees; ANN, Artificial Neural Network; VE, Voting Ensemble.

**Supplementary Table 12.** Inter-explainer comparison of LIME and SHAP for each approach on the **MCI vs CU** dataset

| **Model** | **# Matching Directions (%)**  **[0,65] ꜛ** | **Top 10 Composition Overlap**  **[0,10] ꜛ** | **Mean Absolute Composition Difference**  **[0,100] ꜜ** | **Concordance Index**  **[0,1] ꜛ** | **Number of Leading Predictors for LIME**  **[0,50]** | **Number of Leading Predictors for SHAP**  **[0,50]** |
| --- | --- | --- | --- | --- | --- | --- |
| **LR** | 33 (51%) | **9** | 0.28 | 0.77 | 14 | 13 |
| **SVM** | 33 (51%) | 8 | **0.24** | **0.92** | 16 | 16 |
| **RF** | 31 (48%) | 7 | 0.58 | 0.76 | 13 | 9 |
| **GB** | 29 (45%) | 8 | 0.50 | 0.81 | 9 | 12 |
| **ANN** | 30 (46%) | 7 | 0.57 | 0.78 | 18 | 12 |
| **VE** | **35 (54%)** | 8 | 0.39 | 0.83 | 12 | 15 |
| **Avg.** | **31.83 (49%)** | 7.83 | 0.43 | 0.81 | 13.66 | 12.83 |

*Note.* The ranges of the metrics are displayed beneath the metric name for each column. ꜛ indicates that higher values on the corresponding metric denote better performance. ꜜ indicates that lower values on the corresponding metric denote better performance. Values in bold print identify the best performance by a model for each metric. Abbreviations: LIME, Local Interpretable Model agnostic Explanation; SHAP, SHapley Additive exPlanations; MCI, Mild Cognitive Impairment; CU, Cognitively Unimpaired; LR, Logistic Regression; SVM, Support Vector Machine; RF, Random Forest; GB, Gradient-Boosted trees; ANN, Artificial Neural Network; VE, Voting Ensemble.

**Supplementary Table 13.** Inter-explainer comparison of LIME and SHAP for each approach on the **SCI vs CU** dataset

| **Model** | **# Matching Directions (%)**  **[0,64] ꜛ** | **Top 10 Composition Overlap**  **[0,10] ꜛ** | **Mean Absolute Composition Difference**  **[0,100] ꜜ** | **Concordance Index**  **[0,1] ꜛ** | **Number of Leading Predictors for LIME**  **[0,50]** | **Number of Leading Predictors for SHAP**  **[0,50]** |
| --- | --- | --- | --- | --- | --- | --- |
| **LR** | 28 (44%) | 7 | **0.31** | **0.92** | 20 | 18 |
| **SVM** | **34 (53%)** | **9** | 0.41 | 0.85 | 15 | 12 |
| **RF** | 29 (45%) | 5 | 0.80 | 0.71 | 11 | 9 |
| **GB** | 31 (48%) | 8 | 0.76 | 0.75 | 5 | 12 |
| **ANN** | 28 (44%) | 1 | 1.43 | 0.48 | 17 | 20 |
| **VE** | 33 (52%) | 8 | 0.62 | 0.79 | 12 | 6 |
| **Avg.** | 30.50 (48%) | 6.33 | 0.72 | 0.75 | 13.33 | 12.83 |

*Note.* The ranges of the metrics are displayed beneath the metric name for each column. ꜛ indicates that higher values on the corresponding metric denote better performance. ꜜ indicates that lower values on the corresponding metric denote better performance. Values in bold print identify the best performance by a model for each metric. Abbreviations: LIME, Local Interpretable Model agnostic Explanation; SHAP, SHapley Additive exPlanations; SCI, Subjective Cognitive Impairment; CU, Cognitively Unimpaired; LR, Logistic Regression; SVM, Support Vector Machine; RF, Random Forest; GB, Gradient-Boosted trees; ANN, Artificial Neural Network; VE, Voting Ensemble.

**Supplementary Table 14.** All predictors for the **AD vs CU** dataset across all ML and XAI approach combinations. x indicates that a predictor had > 2.0 composition ratio meaning it accounted for more than 2% of the prediction and is considered a leading predictor.

|  |  | **LIME** | | | | | | **SHAP** | | | | | |
| --- | --- | --- | --- | --- | --- | --- | --- | --- | --- | --- | --- | --- | --- |
| **Predictor** | **Mean Composition Ratio** | **LR** | **SVM** | **RF** | **GB** | **ANN** | **VE** | **LR** | **SVM** | **RF** | **GB** | **ANN** | **VE** |
| **Sex** | 8.64 | x | x | x | x | x | x | x | x | x | x | x | x |
| **BMI** | 0.46 |  |  |  |  |  |  |  |  |  |  |  |  |
| **Hearing_handicap** | 0.23 |  |  |  |  |  |  |  |  |  |  |  |  |
| **Waist_to_hip** | 0.43 |  |  |  |  |  |  |  |  |  |  |  |  |
| **Grip_strength** | 3.28 | x | x | x | x | x | x | x | x | x | x | x | x |
| **Physical_activity** | 1.12 |  |  |  |  |  |  |  |  |  |  |  |  |
| **Olfaction** | 9.77 | x | x | x | x | x | x | x | x | x | x | x | x |
| **Contrast_sensitivity** | 2.18 |  |  | x | x |  |  |  |  | x | x |  |  |
| **Heart_rate** | 0.56 |  |  |  |  |  |  |  |  |  |  |  |  |
| **Waist_circumference** | 1.37 | x |  |  |  | x |  | x |  |  |  |  |  |
| **Pulse_pressure** | 0.74 |  |  |  |  |  |  |  |  |  |  |  |  |
| **Self_rated_balance** | 0.38 |  |  |  |  |  |  |  |  |  |  |  |  |
| **Balance_confidence** | 0.47 |  |  |  |  |  |  |  |  |  |  |  |  |
| **Timed_walk** | 2.98 | x | x |  |  | x | x | x | x | x | x | x | x |
| **Polypharmacy** | 0.95 |  |  |  |  |  |  |  |  |  |  |  |  |
| **Orthostatic_BP_drop** | 0.63 |  |  |  |  |  |  |  |  |  |  |  |  |
| **Unintentional_weight_loss** | 1.07 |  |  |  |  |  |  |  |  |  |  |  |  |
| **Falls** | 0.49 |  |  |  |  |  |  |  |  |  |  |  |  |
| **Osteoarthritis** | 1.59 | x |  |  |  | x |  | x |  |  |  |  |  |
| **Pulmonary_issues** | 1.09 |  |  |  |  | x |  |  |  |  |  |  |  |
| **Sleep_breathing_disorder** | 1.63 |  | x |  | x |  |  |  |  | x | x |  |  |
| **Hypertension** | 0.61 |  |  |  |  |  |  |  |  |  |  |  |  |
| **Atrial_fibrillation** | 0.26 |  |  |  |  |  |  |  |  |  |  |  |  |
| **Heart_attack** | 0.95 |  |  |  |  |  |  |  |  |  |  |  |  |
| **Fainting** | 0.66 |  |  |  |  |  |  |  |  |  |  |  |  |
| **Vertigo** | 1.34 |  |  |  |  | x |  |  |  |  |  | x |  |
| **Hyperlipidemia** | 0.82 |  |  |  |  |  |  |  |  |  |  |  |  |
| **Hypothyroidism** | 1.66 | x | x |  |  | x | x |  |  |  |  | x |  |
| **Osteoporosis** | 0.94 |  | x |  |  |  |  |  |  |  |  |  |  |
| **Ulcers** | 0.74 |  |  |  |  |  |  |  |  |  |  |  |  |
| **Urinary_incontinence** | 0.20 |  |  |  |  |  |  |  |  |  |  |  |  |
| **Cataracts** | 0.99 |  |  |  |  |  |  |  |  |  |  |  |  |
| **Macular_degeneration** | 0.94 |  |  |  |  |  |  |  |  |  |  |  |  |
| **Cancer** | 0.56 |  |  |  |  |  |  |  |  |  |  |  |  |
| **Major_depressive_disorder** | 0.45 |  |  |  |  |  |  |  |  |  |  |  |  |
| **General_anxiety_disorder** | 0.41 |  |  |  |  |  |  |  |  |  |  |  |  |
| **Hemoglobin** | 0.51 |  |  |  |  |  |  |  |  |  |  |  |  |
| **Red_blood_count** | 1.07 |  |  |  |  | x |  |  |  |  |  |  |  |
| **Hematocrit** | 0.47 |  |  |  |  |  |  |  |  |  |  |  |  |
| **Mean_corpuscular_hemoglobin** | 0.82 |  |  | x |  |  |  |  |  |  |  |  |  |
| **Mean_corpuscular_hemoglobin_concentration** | 0.37 |  |  |  |  |  |  |  |  |  |  |  |  |
| **Red_cell_distribution_width** | 1.28 |  |  | x |  |  |  |  |  |  |  |  |  |
| **Lymphocytes_number** | 1.25 |  |  |  |  |  |  |  |  |  |  |  |  |
| **Hemoglobin_A1c** | 1.21 |  |  |  |  |  |  |  |  |  |  |  |  |
| **Sleep_duration** | 0.85 |  |  |  |  |  |  |  |  |  |  |  |  |
| **Sleep_efficiency** | 0.77 |  |  |  |  |  |  |  |  |  |  | x |  |
| **Sleep_disturbances** | 1.35 |  |  | x | x |  |  |  |  |  |  |  |  |
| **Sleeping_meds** | 1.35 |  |  |  |  |  |  |  |  |  |  | x |  |
| **Sleep_latency** | 0.92 |  |  |  |  |  |  |  |  |  |  |  |  |
| **Sleep_daytime_dysfunction** | 0.42 |  |  |  |  |  |  |  |  |  |  |  |  |
| **Everything_an_effort** | 0.54 |  |  |  |  |  |  |  |  |  |  |  |  |
| **Cannot_get_going** | 0.29 |  |  |  |  |  |  |  |  |  |  |  |  |
| **Get_to_bathroom_ADL** | 0.83 |  |  |  |  |  |  |  |  |  |  |  |  |
| **Get_beyond_walking_IADL** | 1.64 |  |  | x |  |  |  |  |  | x | x |  |  |
| **Shopping_IADL** | 3.66 |  | x | x | x |  | x |  |  | x | x |  | x |
| **Prepare_meals_IADL** | 1.64 |  |  | x | x |  |  |  |  | x | x |  |  |
| **Housework_IADL** | 1.71 |  |  | x | x |  |  |  |  |  | x |  |  |
| **Take_medicine_IADL** | 2.33 |  |  | x | x |  | x |  |  | x | x |  |  |
| **Handle_money_IADL** | 3.81 | x | x | x | x | x | x | x |  | x | x | x | x |
| **Physical_health_QOL** | 0.21 |  |  |  |  |  |  |  |  |  |  |  |  |
| **Energy_QOL** | 0.33 |  |  |  |  |  |  |  |  |  |  |  |  |
| **Mood_QOL** | 1.42 |  |  |  |  |  |  |  |  |  |  |  |  |
| **Memory_QOL** | 8.20 | x | x | x | x | x | x | x | x | x | x | x | x |
| **Chores_QOL** | 0.31 |  |  |  |  |  |  |  |  |  |  |  |  |
| **Do_fun_things_QOL** | 1.12 |  |  |  |  |  |  |  |  |  |  |  |  |
| **Self_rated_health** | 0.53 |  |  |  |  |  |  |  |  |  |  |  |  |
| **Self_rated_eyesight** | 0.70 |  |  |  |  |  |  |  |  |  |  |  |  |
| **Self_rated_hearing** | 1.68 |  |  |  |  | x |  |  |  | x |  | x |  |
| **Self_rated_appetite** | 1.09 |  |  |  |  |  |  |  |  | x | x |  |  |
| **Self_rated_mouth_health** | 1.22 |  |  |  |  |  |  |  |  |  |  |  |  |
| **Trouble_swallowing** | 0.82 |  |  |  |  |  |  |  |  |  |  |  |  |
| **Eating_discomfort** | 0.55 |  |  |  |  |  |  |  |  |  |  |  |  |
| **Avoid_eating** | 0.51 |  |  |  |  |  |  |  |  |  |  |  |  |
| **Diabetes** | 0.28 |  |  |  |  |  |  |  |  |  |  |  |  |

*Note.* Details for each of the predictors can be found in Supplementary Table 1. Abbreviations: IADL, instrumental activities of daily living; ADL, basic activities of daily living; QoL, quality of life; BMI, Body Mass Index; BP, Blood Pressure; AD, Alzheimer’s Disease; CU, Cognitively Unimpaired; AUFIC, Area Under the cumulative Feature Importance Curve; LIME, Local Interpretable Model agnostic Explanation; SHAP, SHapley Additive exPlanations; LR, Logistic Regression; SVM, Support Vector Machine; RF, Random Forest; GB, Gradient-Boosted trees; ANN, Artificial Neural Network; VE, Voting Ensemble.

**Supplementary Table 15.** All predictors for the **MCI vs CU** dataset across all ML and XAI approach combinations. x indicates that a predictor had > 2.0 composition ratio meaning it accounted for more than 2% of the prediction and is considered a leading predictor.

|  |  | **LIME** | | | | | | **SHAP** | | | | | |
| --- | --- | --- | --- | --- | --- | --- | --- | --- | --- | --- | --- | --- | --- |
| **Predictor** | **Mean Composition Ratio** | **LR** | **SVM** | **RF** | **GB** | **ANN** | **VE** | **LR** | **SVM** | **RF** | **GB** | **ANN** | **VE** |
| **Sex** | 9.20 | x | x | x | x | x | x | x | x | x | x | x | x |
| **Hearing_handicap** | 0.98 |  |  |  |  |  |  |  |  |  |  |  |  |
| **Waist_to_hip** | 0.46 |  |  |  |  |  |  |  |  |  |  |  |  |
| **Grip_strength** | 5.48 | x | x | x | x | x | x | x | x | x | x | x | x |
| **Physical_activity** | 0.27 |  |  |  |  |  |  |  |  |  |  |  |  |
| **Olfaction** | 2.11 |  | x |  |  | x |  |  | x |  |  | x | x |
| **Contrast_sensitivity** | 0.34 |  |  |  |  |  |  |  |  |  |  |  |  |
| **Heart_rate** | 0.40 |  |  |  |  |  |  |  |  |  |  |  |  |
| **Waist_circumference** | 0.49 |  |  |  |  |  |  |  |  |  |  |  |  |
| **Pulse_pressure** | 4.56 | x | x | x | x | x | x | x | x | x | x | x | x |
| **Self_rated_balance** | 0.29 |  |  |  |  |  |  |  |  |  |  |  |  |
| **Balance_confidence** | 0.28 |  |  |  |  |  |  |  |  |  |  |  |  |
| **Timed_walk** | 0.48 |  |  |  |  |  |  |  |  |  |  |  |  |
| **Polypharmacy** | 0.40 |  |  |  |  |  |  |  |  |  |  |  |  |
| **BM** | 0.53 |  |  |  |  |  |  |  |  |  |  |  |  |
| **Sleep_duration** | 0.72 |  |  |  |  |  |  |  |  |  |  |  |  |
| **Sleep_efficiency** | 2.38 | x | x |  |  |  | x | x | x |  | x |  | x |
| **Sleep_disturbances** | 1.15 |  |  | x |  |  |  |  |  |  |  |  |  |
| **Sleeping_meds** | 1.91 |  | x |  |  | x |  |  | x |  |  |  | x |
| **Sleep_latency** | 1.35 | x |  |  |  | x |  | x |  |  |  |  |  |
| **Sleep_daytime_dysfunction** | 0.47 |  |  |  |  |  |  |  |  |  |  |  |  |
| **Orthostatic_BP_drop** | 1.43 |  |  | x |  |  |  |  |  |  | x |  |  |
| **Everything_an_effort** | 1.27 |  |  |  |  |  |  |  |  |  |  |  |  |
| **Cannot_get_going** | 1.02 |  |  |  |  |  |  |  |  |  |  |  |  |
| **Get_to_bathroom_ADL** | 0.42 |  |  |  |  |  |  |  |  |  |  |  |  |
| **Physical_health_QOL** | 1.17 |  |  | x | x |  |  |  |  |  |  |  |  |
| **Energy_QOL** | 0.43 |  |  |  |  |  |  |  |  |  |  |  |  |
| **Mood_QOL** | 1.25 |  |  | x |  |  |  |  |  |  |  |  |  |
| **Memory_QOL** | 14.20 | x | x | x | x | x | x | x | x | x | x |  | x |
| **Chores_QOL** | 0.42 |  |  |  |  |  |  |  |  |  |  |  |  |
| **Do_fun_things_QOL** | 0.86 |  |  |  |  |  |  |  |  |  |  |  |  |
| **Self_rated_health** | 3.03 | x |  | x | x | x | x | x |  | x | x | x | x |
| **Falls** | 2.28 | x | x |  |  | x | x | x | x |  |  | x | x |
| **Self_rated_eyesight** | 1.16 |  |  | x |  |  |  |  |  | x |  |  |  |
| **Self_rated_hearing** | 1.30 | x |  |  |  |  |  | x |  |  | x |  |  |
| **Self_rated_appetite** | 0.67 |  |  |  |  |  |  |  |  |  |  |  |  |
| **Trouble_swallowing** | 1.83 |  |  |  |  |  |  |  | x | x | x |  | x |
| **Self_rated_mouth_health** | 0.30 |  |  |  |  |  |  |  |  |  |  |  |  |
| **Eating_discomfort** | 0.59 |  |  |  |  | x |  |  |  |  |  |  |  |
| **Avoid_eating** | 0.40 |  |  |  |  |  |  |  |  |  |  |  |  |
| **Osteoarthritis** | 1.23 |  | x |  |  | x |  |  | x |  |  | x |  |
| **Pulmonary_issues** | 0.47 |  |  |  |  |  |  |  |  |  |  |  |  |
| **Sleep_breathing_disorder** | 0.90 |  |  |  |  |  |  |  |  |  |  |  |  |
| **Hypertension** | 0.60 |  |  |  |  |  |  |  |  |  |  |  |  |
| **Atrial_fibrillation** | 0.82 |  |  |  |  |  |  |  |  |  |  |  |  |
| **Transient_ischemic_attack** | 0.36 |  |  |  |  |  |  |  |  |  |  |  |  |
| **Fainting** | 2.89 | x | x |  |  | x | x | x | x |  |  | x | x |
| **Vertigo** | 2.26 |  | x | x | x | x | x |  | x |  | x | x | x |
| **Diabetes** | 1.34 |  |  |  |  |  |  |  |  |  | x |  |  |
| **Hyperlipidemia** | 1.95 | x |  |  |  | x |  | x | x |  |  | x | x |
| **Hypothyroidism** | 0.66 |  |  |  |  |  |  |  |  |  |  | x |  |
| **Osteoporosis** | 0.53 |  |  |  |  |  |  |  |  |  |  |  |  |
| **Ulcers** | 0.70 |  |  |  |  |  |  |  |  |  |  |  |  |
| **Chronic_constipation** | 0.63 |  |  |  |  | x |  |  |  |  |  |  |  |
| **Urinary_incontinence** | 1.73 |  | x |  |  | x | x |  | x |  |  | x |  |
| **Cataracts** | 0.25 |  |  |  |  |  |  |  |  |  |  |  |  |
| **Cancer** | 0.65 |  |  |  |  |  |  |  |  |  |  |  |  |
| **Major_depressive_disorder** | 1.41 | x | x |  |  |  |  | x | x |  |  |  |  |
| **General_anxiety_disorder** | 0.78 |  |  |  |  |  |  |  |  |  |  |  |  |
| **Hemoglobin** | 0.57 |  |  | x |  |  |  |  |  |  |  |  |  |
| **Red_blood_count** | 1.27 |  | x |  | x | x |  |  |  |  |  |  |  |
| **Hematocrit** | 0.61 |  |  |  |  |  |  |  |  |  |  |  |  |
| **Mean_corpuscular_volume** | 0.44 |  |  |  |  |  |  |  |  |  |  |  |  |
| **Lymphocytes_number** | 5.92 | x | x | x | x |  | x |  |  | x | x |  | x |
| **hba1c** | 2.45 | x | x |  |  | x | x | x | x | x |  |  | x |

*Note.* Details for each of the predictors can be found in Supplementary Table 1. Abbreviations: IADL, instrumental activities of daily living; ADL, basic activities of daily living; QoL, quality of life; HbA1c, glycated hemoglobin.d; BMI, Body Mass Index; BP, Blood Pressure; MCI, Mild Cognitive Impairment; CU, Cognitively Unimpaired; AUFIC, Area Under the cumulative Feature Importance Curve; LIME, Local Interpretable Model agnostic Explanation; SHAP, SHapley Additive exPlanations; LR, Logistic Regression; SVM, Support Vector Machine; RF, Random Forest; GB, Gradient-Boosted trees; ANN, Artificial Neural Network; VE, Voting Ensemble.

**Supplementary Table 16.** All predictors for the **SCI vs CU** dataset across all ML and XAI approach combinations. x indicates that a predictor had > 2.0 composition ratio meaning it accounted for more than 2% of the prediction and is considered a leading predictor.

|  |  | **LIME** | | | | | | **SHAP** | | | | | |
| --- | --- | --- | --- | --- | --- | --- | --- | --- | --- | --- | --- | --- | --- |
| **Predictor** | **Mean Composition Ratio** | **LR** | **SVM** | **RF** | **GB** | **ANN** | **VE** | **LR** | **SVM** | **RF** | **GB** | **ANN** | **VE** |
| **Sex** | 0.78 |  |  |  |  | x |  |  |  |  |  |  |  |
| **BMI** | 0.92 |  |  |  |  |  |  |  |  |  |  |  |  |
| **Hearing_handicap** | 1.27 |  |  |  |  |  |  |  |  |  | x |  |  |
| **Waist_to_hip** | 1.76 | x |  |  |  | x |  | x | x |  |  | x |  |
| **Grip_strength** | 3.16 | x | x |  |  |  | x | x | x | x | x |  | x |
| **Physical_activity** | 0.58 |  |  |  |  | x |  |  |  |  |  |  |  |
| **Olfaction** | 1.16 |  |  |  |  | x |  |  |  |  |  | x |  |
| **Heart_rate** | 2.39 | x | x |  |  | x |  | x | x |  |  |  |  |
| **Waist_circumference** | 2.74 | x | x |  |  |  | x | x | x | x |  | x |  |
| **Pulse_pressure** | 1.26 |  |  |  |  | x |  |  |  |  |  |  |  |
| **Self_rated_balance** | 1.20 |  |  |  |  |  |  |  |  |  |  | x |  |
| **Balance_confidence** | 1.13 |  |  |  |  |  |  |  |  |  |  |  |  |
| **Timed_walk** | 1.78 | x | x |  |  | x |  | x | x |  |  |  |  |
| **Polypharmacy** | 1.51 | x |  | x |  |  |  |  |  |  |  | x |  |
| **Sleep_duration** | 0.43 |  |  |  |  |  |  |  |  |  |  |  |  |
| **Sleep_efficiency** | 0.79 |  |  |  |  |  |  |  |  |  |  |  |  |
| **Sleep_disturbances** | 0.63 |  |  |  |  |  |  |  |  |  |  |  |  |
| **Sleeping_meds** | 2.25 | x |  | x | x |  | x | x |  |  | x | x |  |
| **Sleep_latency** | 0.54 |  |  |  |  |  |  |  |  |  |  |  |  |
| **Sleep_daytime_dysfunction** | 0.85 |  |  |  |  |  |  |  |  |  |  | x |  |
| **Orthostatic_BP_drop** | 2.02 | x | x | x |  |  |  |  |  |  | x | x |  |
| **Unintentional_weight_loss** | 1.30 |  |  |  |  |  |  |  |  | x | x |  | x |
| **Everything_an_effort** | 0.72 |  |  |  |  |  |  |  |  |  |  |  |  |
| **Cannot_get_going** | 0.45 |  |  |  |  |  |  |  |  |  |  |  |  |
| **Get_to_bathroom_ADL** | 2.97 | x | x |  |  |  | x | x | x | x |  | x |  |
| **Housework_IADL** | 0.78 |  |  |  |  |  |  |  |  |  |  |  |  |
| **Physical_health_QOL** | 0.69 |  |  |  |  | x |  |  |  |  |  |  |  |
| **Energy_QOL** | 1.31 |  |  | x |  |  |  |  |  | x |  |  |  |
| **Mood_QOL** | 1.02 |  |  |  |  |  |  |  |  |  |  |  |  |
| **Memory_QOL** | 9.77 | x | x | x | x | x | x | x | x | x | x |  | x |
| **Chores_QOL** | 0.51 |  |  |  |  |  |  |  |  |  |  |  |  |
| **Do_fun_things_QOL** | 1.24 |  |  |  |  |  |  |  |  |  | x | x |  |
| **Self_rated_health** | 0.76 |  |  |  |  | x |  |  |  |  |  |  |  |
| **Falls** | 2.49 | x | x |  |  |  | x | x | x |  |  |  |  |
| **Self_rated_eyesight** | 0.55 |  |  |  |  |  |  |  |  |  |  |  |  |
| **Self_rated_hearing** | 1.22 |  |  |  |  |  |  |  |  |  |  |  |  |
| **Self_rated_appetite** | 1.14 |  |  | x |  |  |  |  |  |  |  |  |  |
| **Self_rated_mouth_health** | 1.38 |  |  | x | x |  |  |  |  |  | x |  |  |
| **Trouble_swallowing** | 0.91 |  |  |  |  |  |  |  |  |  | x |  |  |
| **Eating_discomfort** | 1.09 |  |  |  |  |  |  |  |  |  |  | x |  |
| **Avoid_eating** | 0.54 |  |  |  |  |  |  |  |  |  |  |  |  |
| **Osteoarthritis** | 0.99 |  |  |  |  | x |  |  |  |  |  | x |  |
| **Pulmonary_issues** | 1.77 | x | x |  |  |  | x | x | x |  |  |  |  |
| **Sleep_breathing_disorder** | 0.59 |  |  |  |  |  |  |  |  |  |  |  |  |
| **Hypertension** | 1.55 | x |  |  |  |  |  | x |  |  |  | x |  |
| **Atrial_fibrillation** | 0.60 |  |  |  |  |  |  |  |  |  |  |  |  |
| **Peripheral_vascular_disease** | 0.95 |  |  |  |  |  |  |  |  |  |  |  |  |
| **Fainting** | 0.78 |  |  |  |  |  |  |  |  |  |  | x |  |
| **Vertigo** | 1.64 | x | x |  |  |  |  | x | x |  |  | x |  |
| **Hyperlipidemia** | 0.75 |  |  |  |  |  |  |  |  |  |  |  |  |
| **Hypothyroidism** | 1.96 | x | x | x |  |  |  | x |  |  |  | x |  |
| **Osteoporosis** | 1.42 | x |  |  |  | x | x | x |  |  |  |  |  |
| **Ulcers** | 0.88 |  |  |  |  | x |  |  |  |  |  |  |  |
| **Irritable_bowel_syndrome** | 0.72 |  |  |  |  |  |  |  |  |  |  |  |  |
| **Urinary_incontinence** | 0.95 |  |  |  |  | x |  |  |  |  |  |  |  |
| **Cataracts** | 1.00 |  |  |  |  |  |  | x |  |  |  | x |  |
| **Cancer** | 1.32 |  |  |  |  | x |  |  |  |  |  | x |  |
| **Major_depressive_disorder** | 1.23 |  | x |  |  | x |  |  |  |  |  |  |  |
| **General_anxiety_disorder** | 1.71 | x | x |  |  |  | x |  |  |  |  |  |  |
| **Red_blood_count** | 0.81 |  |  |  |  |  |  |  |  |  |  | x |  |
| **White_blood_count** | 3.01 | x | x |  |  | x | x | x | x | x | x |  | x |
| **Neutrophils_number** | 5.21 |  |  | x | x |  | x |  |  | x | x |  | x |
| **Lymphocytes_number** | 8.13 | x | x | x | x |  | x | x | x | x | x |  | x |
| **Hemoglobin_A1c** | 1.73 | x |  | x |  |  |  | x |  |  |  | x |  |

*Note.* Details for each of the predictors can be found in Supplementary Table 1. Abbreviations: IADL, instrumental activities of daily living; ADL, basic activities of daily living; QoL, quality of life; BMI, Body Mass Index; BP, Blood Pressure; SCI, Subjective Cognitive Impairment; CU, Cognitively Unimpaired; AUFIC, Area Under the cumulative Feature Importance Curve; LIME, Local Interpretable Model agnostic Explanation; SHAP, SHapley Additive exPlanations; LR, Logistic Regression; SVM, Support Vector Machine; RF, Random Forest; GB, Gradient-Boosted trees; ANN, Artificial Neural Network; VE, Voting Ensemble.

# References

Washburn, R. A., Smith, K. W., Jette, A. M., and Janney, C. A. (1993). The Physical Activity Scale for the Elderly (PASE): Development and evaluation. Journal of Clinical Epidemiology, 46(2), 153–162. https://doi.org/10.1016/0895-4356(93)90053-4

Powell, L. E., and Myers, A. M. (1995). The Activities-specific Balance Confidence (ABC) Scale. The Journals of Gerontology Series A: Biological Sciences and Medical Sciences, 50A(1), M28–M34. https://doi.org/10.1093/gerona/50a.1.m28

Ventry, I. M., and Weinstein, B. E. (1982). The hearing handicap inventory for the elderly: A new tool. Ear and Hearing, 3(3), 128–134. https://doi.org/10.1097/00003446-198205000-00006

Menon, C., Westervelt, H. J., Jahn, D. R., Dressel, J. A., and O’Bryant, S. E. (2013). Normative performance on the Brief Smell Identification Test (BSIT) in a multi-ethnic bilingual cohort: A Project FRONTIER study. The Clinical Neuropsychologist, 27(6), 946–961. https://doi.org/10.1080/13854046.2013.796406

Buysse, D. J., Reynolds, C. F., Monk, T. H., Berman, S. R., and Kupfer, D. J. (1989). The Pittsburgh Sleep Quality Index: A new instrument for psychiatric practice and research. Psychiatry Research, 28(2), 193–213. https://doi.org/10.1016/0165-1781(89)90047-4

Bohn, L., Drouin, S. M., McFall, G. P., Rolfson, D. B., Andrew, M. K., and Dixon, R. A. (2023). Machine learning analyses identify multi-modal frailty factors that selectively discriminate four cohorts in the Alzheimer’s Disease Spectrum: A Compass-ND Study. BMC Geriatrics, 23(1). https://doi.org/10.1186/s12877-023-0454
